# Seven steps to mapping health service provision: lessons learned from mapping services for adults with Attention-Deficit/Hyperactivity Disorder (ADHD) in the UK

**DOI:** 10.1186/s12913-019-4287-7

**Published:** 2019-07-09

**Authors:** Anna Price, Astrid Janssens, Susan Dunn-Morua, Helen Eke, Philip Asherson, Tony Lloyd, Tamsin Ford

**Affiliations:** 1 University of Exeter Medical School, St Luke’s Campus, Exeter, EX1 2LU UK; 2 Department of Public Health, University of Southern Denmark, J. B. Winsløws Vej 9B, DK-5000 Odense C, Denmark; 3 AADD-UK, 3 Eastcroft, Blagdon, North Somerset, BS40 7RT UK; 4 Kings College London, Institute of Psychiatry Psychology and Neuroscience, De Crespigny Park, Denmark Hill, London, SE5 8AF UK; 5 UK Adult ADHD Network, London, UK; 6 ADHD Foundation, 151 Dale Street, Liverpool, L2 2AH UK

**Keywords:** (3–10) mapping, Survey, ADHD, Transition, Health service provision

## Abstract

**Background:**

ADHD affects some individuals throughout their lifespan, yet service provision for adults in the United Kingdom (UK) is patchy. Current methods for mapping health service provision are resource intensive, do not map specialist ADHD teams separately from generic mental health services, and often fail to triangulate government data with accounts from service users and clinicians. Without a national audit that maps adult ADHD provision, it is difficult to quantify current gaps in provision and make the case for change. This paper describes the development of a seven step approach to map adult ADHD service provision in the UK.

**Methods:**

A mapping method was piloted in 2016 and run definitively in 2018. A seven step method was developed: 1. Defining the target service 2. Identifying key informants 3. Designing the survey 4. Data collection 5. Data analysis 6. Communicating findings 7. Hosting/updating the service map. Patients and members of the public (including clinicians and commissioners) were involved with design, data collection and dissemination of findings.

**Results:**

Using a broad definition of adult ADHD services resulted in an inclusive list of identified services, and allowed the definition to be narrowed to National Health Service (NHS) funded specialist ADHD services at data analysis, with confidence that few relevant services would be missed. Key informants included patients, carers, a range of health workers, and commissioners. A brief online survey, written using lay terms, appeared acceptable to informants. Emails sent using national organisations’ mailing lists were the most effective way to access informants on a large scale. Adaptations to the methodology in 2018 were associated with 64% more responses (2371 vs 1446) collected in 83% less time (5 vs 30 weeks) than the pilot. The 2016 map of adult ADHD services was viewed 13,688 times in 17 weeks, indicating effective communication of findings.

**Conclusion:**

This seven step pragmatic method was effective for collating and communicating national service data about UK adult ADHD service provision. Patient and public involvement and engagement from partner organisations was crucial throughout. Lessons learned may be transferable to mapping service provision for other health conditions and in other locations.

**Electronic supplementary material:**

The online version of this article (10.1186/s12913-019-4287-7) contains supplementary material, which is available to authorized users.

## Background

Attention-deficit/hyperactivity disorder (ADHD) is a neurodevelopmental disorder that affects a significant proportion of individuals across their lifespan, for which there are effective, evidence based treatments [1]. Mental health service provision in higher income countries is separate for children and adults, and the transition often occurs at a key developmental stage [2, 3]. Epidemiological studies show an estimated prevalence of ADHD of 5–6% in children, and 3–4% in adults [4–7]. The number of young people with ADHD graduating from children’s services has increased rapidly, because of a rise in childhood prescription rates, [8] and studies have found that many young people with ADHD are likely to need continued care into adulthood [9–11]. Therefore providing a supported transition for this group in line with clinical guidelines is important [12, 13]. However, provision for adults with ADHD remains relatively scarce across the world [14] and is known to be patchy and difficult to access in the United Kingdom (UK) [15].

Atlases of Health, which map international health service provision using government and expert sources, are well-established tools designed to provide objective and reliable information on healthcare service provision [16–18]. The European Service Mapping Schedule [19] is a survey instrument for the description and classification of mental health services. It was adapted by Signorini et al. [20], and used to survey child psychiatry representatives on the characteristics of child and adolescent mental health services (CAMHS) across the European Union (EU). While valuable, these tools often fail to triangulate government and expert reports of service provision with the experience of service users and clinicians in practice. The general focus also means that they may not capture condition specific information.

The Atlas of Variation series [21] uses routinely available data and consultation with clinical experts, to provide government reports (with maps, charts and time-series data) on provision and patient outcomes for a selection of health topics [22]. However, to date, ADHD services and outcomes have not been mapped. The information provided is also highly complex, difficult for lay readers to understand and does not include accounts from service users and clinicians. Independent regulators such as the Care Quality Commission use inspection methodology including consultations with staff and service users and observing clinical practice to provide detailed reports on the state of care [23]. Findings are reported in a format that is accessible to a range of stakeholders. However, this is a resource intensive process, and most specialisms are not identified separately from community mental health, which makes it impossible to learn about adult ADHD service provision from these reports [23]. Without a national audit aimed specifically at mapping adult ADHD provision, drawing on a range of stakeholder sources, it is difficult to quantify and address current gaps in healthcare and make the case for appropriate change.

In a recent survey of every National Health Service (NHS) mental health trust in England, senior health professionals were asked to provide information on transition protocols, pathways and commissioned services for ADHD [24]. Over two thirds of NHS Trusts responded (68%). The survey was designed to be completed by a senior healthcare professional within the trust and it is unknown whether non-response from 17 trusts reflects reluctance to report on gaps in services or other reasons for non-response such a lack of time or personnel. Less than half of the responding mental health trusts in England offered specialist provision for adults with ADHD and less than a third had specific commissioning arrangements for this group [24]. In a separate survey, all healthcare professionals working in child and adult health services in the East Midlands region of England, were asked about transitional health services for young people with ADHD [25]. The overwhelming majority of respondents reported a lack of provision [25]. Despite a relatively low response rate (19%), surveying all staff resulted in responses from a variety of professionals working with people with ADHD including psychiatrists, managers, nurses and paediatricians. This method, although more resource intensive and limited to a smaller geographic area, included perspectives of clinicians working daily with patients. It is possible however, that they will not have daily experience of service provision in practice, while the pressures of managing a resource strapped service may conflict with straightforward reporting.

Our recent systematic review of qualitative research about transition into adult ADHD services found that a lack of available information about adult ADHD services created difficulties in accessing treatment [26]. People with ADHD reported they did not know where to access treatment [27], while some clinicians reported difficulties in finding an adult service to refer patients on to [28]. This work indicates the importance of information about where services for ADHD are, what they offer and how to access them. Methods used to map ADHD services need to collect data that is relevant and accessible to patients and clinicians as well as service providers and commissioners. Different stakeholders are likely to have different perspectives on what is as well as what needs to be available and it would be interesting to explore differences between provider’s reports on service availability and patient experiences of provision.

To extend and expand the findings from previous research [20, 24, 25], we piloted and refined a multi-informant, multi-source methodology to map adult ADHD provision in the UK. This paper describes the seven step approach that we developed. The methods used are intended to meet current needs for national service data specific to ADHD and to enable a comparison of differences in reported information by different stakeholder groups.

## Methods

**Fig. 1:**
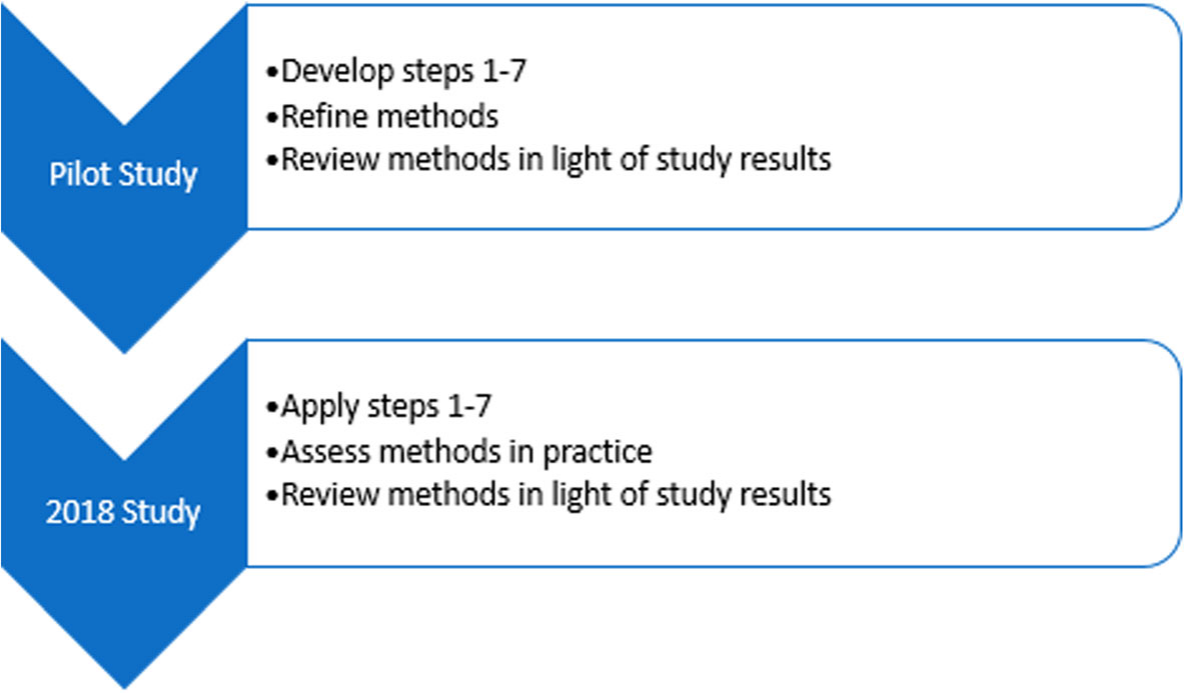
Process of refining methods

**Fig. 2:**
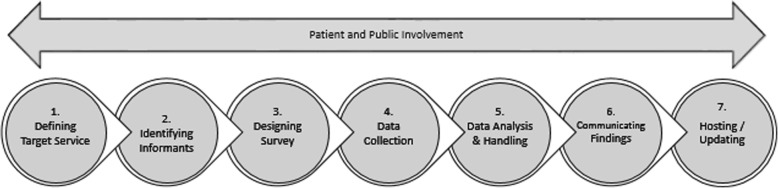
Seven steps to map a service

The mapping methodology was piloted in 2016, followed by a definitive study in 2018: the mapping findings are reported in full elsewhere [29]. An iterative process of trialling and reviewing methods (Fig. 1), led the development of the seven step mapping method described in the current paper (Fig. 2).

**Fig. 3:**
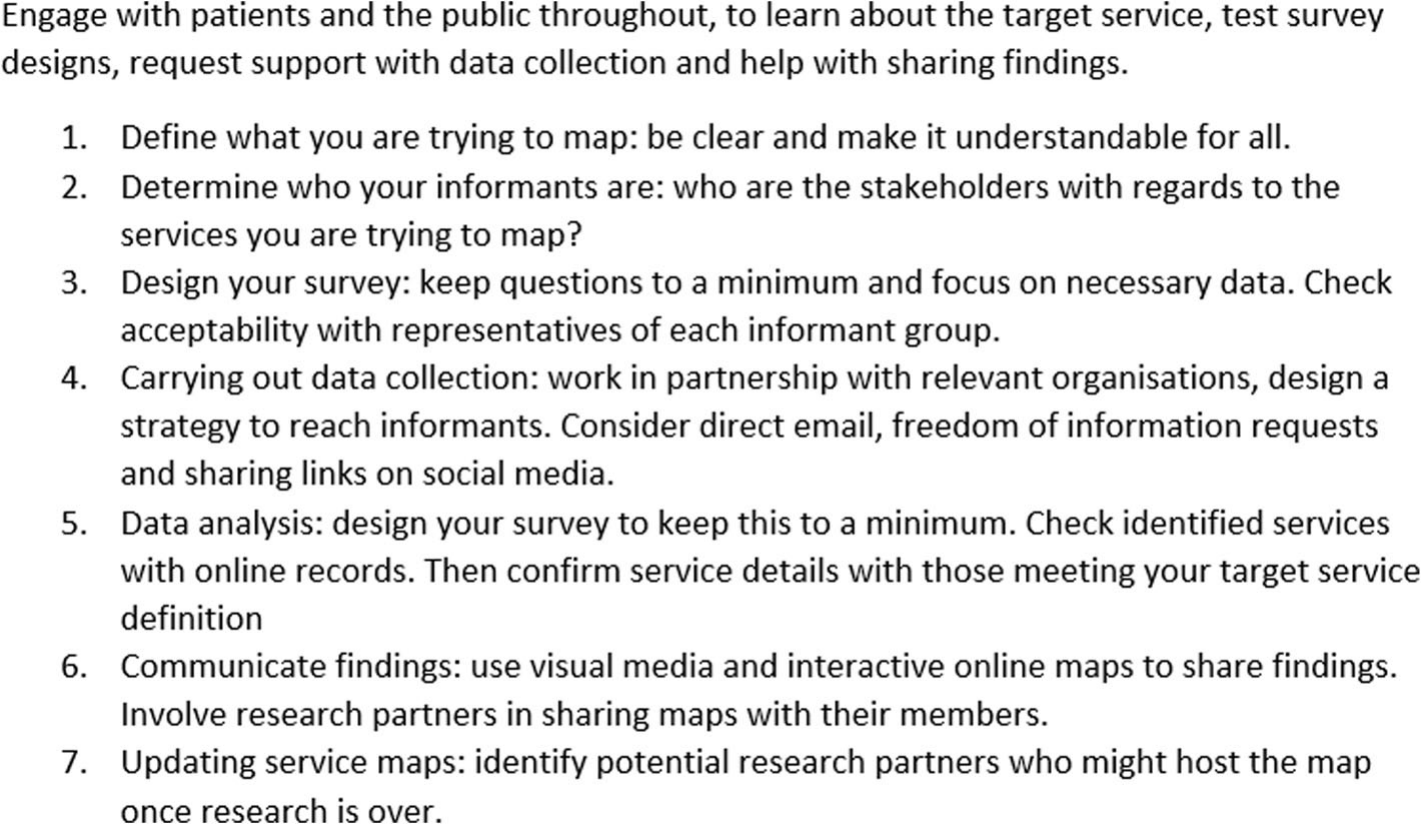
Steps identified in running a mapping study

For a detailed seven step description of piloting work and adaptations involved in the development of this method see Additional file 1. For a summary of the steps identified, see Fig. 3.

### Patient and public involvement

People with ADHD and their families, health workers, and commissioners were involved with design, data collection, and dissemination of research findings. An advisory group of parents of young people with mental health difficulties, including ADHD, guided the iterative development of methods. Clinical and ADHD focussed organisations supported distribution of the surveys and communication of findings.

### Defining target services

Services to be included were broadly defined as “*any mental health service for people with ADHD aged 18 and above*” located in the UK. In the *2018 study*, notes were added to clarify that this could include any “*specialist doctor or team, mental health team, clinic, charity or support group that treats or supports adults with ADHD*” (see Additional files 2 and 3). Following data collection, a second narrower definition was used to create a more focussed list of either: adult NHS specialist, private and charitable services, with a focus on treating ADHD or neurodevelopmental conditions (*pilot*); or dedicated adult ADHD services funded by the NHS (*2018 study*).

### Sample

As Table 1 illustrates, informants were purposively sampled from a range of key stakeholder groups (service users, clinicians, and commissioners) via multiple methodologies and sampling frames.

**Table 1:** Mapping study sample

Non-probabilistic sampling frame	Survey method	Data collection format	Stakeholders surveyed
Total population	Survey of NHS commissioning organisations	• Emails sent to NHS England commissioners^a^ • FOI requests sent to NHS England^a,b^, Scotland^b^, Wales^b^ and Northern Ireland^b^ commissioning bodies	Commissioners
Convenience	Online survey	• email with link sent via organisational mailing lists^a,b,c^ • link included in organisational newsletters^a,b,c^ • link shared on organisations’ websites^a,b,c^ • link shared via Twitter and other social media^a,b^	Health workers, service users, commissioners, and others

*NHS* National Health Service, *FOI* freedom of information, *ADHD* attention-deficit/hyperactivity disorder, *AMHS* adult mental health service,^a^Pilot study, ^b^2018 study, ^c^for a list of partner organisations see Additional file 4

### Data collection

Data on services was collected using a brief survey. Surveys were either, tailored by respondent type and made up of 9–15 questions (*pilot*) (see Additional file 2), or suitable for all respondent types and made up of 5–9 questions (*2018 survey)* (see Additional file 3). The survey collected basic demographic information, including the role or ‘identity’ of informants, with a drop down list of options including ‘other’ (see Additional file 2). Informants were asked to provide details of services they knew about for adults with ADHD. In 2018 respondents were asked to view a pre-populated list of services identified in the pilot, before being asked to provide details of any other services. They were also asked to confirm whether they knew of someone who had used that service. Surveys were distributed using a variety of methods (see Table 1). In 2018, data collection was planned in advance with research partners (see Additional file 4), and informants were given the opportunity to indicate additional roles, and asked to indicate whether they, or anyone they knew, had experience of using an identified service for treatment/support of adult ADHD.

### Analysis

Descriptive statistics summarised the numbers of and geographic locations of respondents by informant group. An open source geographic information system, QGIS 2.18 [30], was used to display the balance of responses by UK region and stakeholder group.

#### Data cleaning

Raw data on services provided by informants, were uploaded into Excel and matched against existing online information by the lead researcher, using search terms derived from the informant’s description, their geographic location, and other terms relevant to search such as “*ADHD, neurodevelopmental,* and *health service*”. Where service details could not be matched against online data, they were independently checked. Unmatched service details were checked a minimum of three times before being categorised as unidentifiable.

#### Service checking

Details of identified services such as location, service name, and the provider organisation were verified against online information (for example, via health provider or support group websites, or through service leaflets) by a second researcher. A list of services was created and categorised by type, provider organisation and location. A record was kept of which informants had identified which service.

Identified services were divided into two groups, with group one including all identified services, while group two was restricted to either: specialist ADHD services, including adult NHS specialist, private and charitable services with a focus on treating ADHD; or neurodevelopmental conditions *(pilot);* or dedicated adult ADHD services, funded by the NHS (*2018 survey*).

For adult NHS specialist services, details of the treatment and support provided was checked via a brief questionnaire completed by the relevant health provider: phone or email contact (*pilot*); or via freedom of information (FOI) requests (*pilot and 2018 survey*) (Additional file 5). FOIs give individuals the right to access recorded information held by public sector organisations, and allow administrators to prioritise the request [31].

#### Reliability and validity

Non-probabilistic sampling methods, which were cost effective and enabled rapid data collection, were chosen to meet survey aims of covering a wide geographic area, minimising missing data, and including multiple informants. Data collection methods were developed iteratively to ensure information was gathered from a range of informants and on as many relevant services as possible, with the aim that the resultant map of services would provide reliable and valid information. In the *2018 survey*, a pragmatic target of collecting data from a minimum of 51 informants per UK region was identified.

### Communicating findings

Interactive maps of identified services were created using Google My Maps, to communicate data on identified services with stakeholders [32, 33].

## Results

### Defining target service

Dividing identified services into two groups led to two lists of services. The first was a comprehensive and inclusive index of the wide range of public, private and voluntary services in the UK reported by informants, where adults with ADHD could access treatment/support. The second was a map of NHS funded specialist ADHD services, with details about treatments available.

In the pilot study, respondent provided information on treatment/support available at services was unreliable as conflicting details were given for the same service, and these questions were removed in the 2018 study. Checking specifications of provision via the service/relevant NHS trust at the service checking stage provided more consistent data. The inclusion of a question in 2018, asking informants if they knew of someone who had used the service, made it possible to distinguish services which informants had direct knowledge of from those they had just heard of.

### Identifying informants

Targeting a range of key stakeholders made it possible to investigate differences in service knowledge between groups. During the pilot, ongoing qualitative research [34] highlighted the important role of primary care clinicians as gatekeepers of specialist services, and data collection was adjusted part-way through the pilot to include general practitioners (GPs).

Free text responses of ‘*other’* to the identity question in the pilot provided data which was used to populate the list of stakeholder identity options in 2018 (see Table 2). The pilot received 224 (15%) responses of ‘*other’*, compared with 86 (4%) in 2018, indicating this was a more acceptable list of pre-populated options.

**Table 2:** ‘Identity’ categories used in the pilot and adapted/added to for the 2018 survey

Pilot	2018
Young person (from 14 up to 17 years old)	Young person with ADHD (up to 17 years old)
Young adult (18 or older)	Adult with ADHD (aged 18+)
A parent/carer of a person with ADHD	Parent/carer/partner of someone with ADHD
A clinician working with young people and/or adults with ADHD	In an ADHD support role (e.g. voluntary, support work or training)
Paediatrician	Paediatrician
Psychiatrist	Psychiatrist
General Practitioner	General Practitioner
Other (please specify)	Clinical Psychologist
	Educational Practitioner (e.g. support worker, Teacher, Behavioural Support, Ed Psych, EWO)
	Nurse
	Manager
	Allied Health professional
	Researcher or Academic
	Administrator
	Clinical Commissioner
	Other (please specify)

*ADHD* attention-deficit/hyperactivity disorder, *Ed Psych* educational psychologist, *EWO* education welfare officer

In 2018, 1010 (47%) of informants identified themselves as having two or more roles.

### Designing the survey

Use of online survey methodology and a short questionnaire format appeared acceptable. Surveys were designed using lay terms to be accessible to all informants. The 2018 survey took respondents a median of 3 min to complete, and achieved 2371 responses with 79% completing all relevant questions.

Designing a single questionnaire for all informant groups in 2018 simplified data collection, as stakeholders could be sent a single link. It reduced subsequent data cleaning and enabled use of analytic tools built into the hosting software to rapidly identify areas/key groups where responses were low. Use of a pre-populated list of identified services in 2018 appeared acceptable, and reduced data cleaning.

### Data collection

The 2018 strategy of planning data collection in advance with research partners, with a primary focus on emails, was associated with 64% more responses (1446 compared with 2371) in 83% less time (5 weeks compared with 30) when compared with the pilot.

Distributing links via emails sent from national organisations’ mailing lists appeared to be the most effective dissemination tool, with high response numbers for stakeholders where this strategy was used (see Table 3). Response numbers were relatively low when direct email was not possible, despite survey promotion via organisational newsletters and social media.

**Table 3:** Pilot and 2018 survey data collection strategies used; response numbers by stakeholder group

Stakeholders	Pilot		2018 Survey	
	Strategies	Responses	Strategies	Responses
Psychiatrists	1^a^,4,5	380	1^a^,4,5	530
Paediatricians	3,5	104	3,5	74
GPs	2,5	200	1^a^,5	387
Health Professionals	2	116	1^a^	306
Service Users	3,5	477	3,5	455

1 = email via national organisation’s mailing list, 2 = email via regional organisation, 3 = Promotion via organisation’s newsletter/website, 4 = promotion via conference, 5 = promotion via social media, ^a^ = strategy associated with high response numbers

Use of social media, in particular Twitter, appeared to raise awareness of the survey. During the 2018 survey, linking with relevant organisations resulted in high levels of engagement on Twitter, with 44,000 tweet impressions, and 101 survey link clicks. It is unknown how tweet impressions relate to survey response numbers.

The first approach to contacting commissioning organisations, via direct email, resulted in a low response rate of 9%. Subsequent use of FOI requests resulted in excellent response rates of 80–90% (see Table 4).

**Table 4:** Response rates from commissioning organisations, with data collection strategies used

Commissioning Organisations	Strategy	Response numbers (response rate %)	
		Pilot	2018 Survey
CCGs (England)	Email^a^	19 (9%)	N/A
CCGs (England)	FOI ^b^	169 (80%)	190 (89)
Health Boards (Scotland)	FOI ^b^	N/A	12 (86)
Health Boards (Wales)	FOI^b^	N/A	6 (86)
Health and Social Care Trust (Northern Ireland)	FOI^b^	N/A	5 (100)
Total		169 (80%)	213 (90%)

*CCG* Clinical commissioning group,^a^an email was sent with a survey link or, if requested, with the questionnaire attached, ^b^an official Freedom of Information (FOI) request sent with the questionnaire attached

#### Balance of responses

**Fig. 4:**
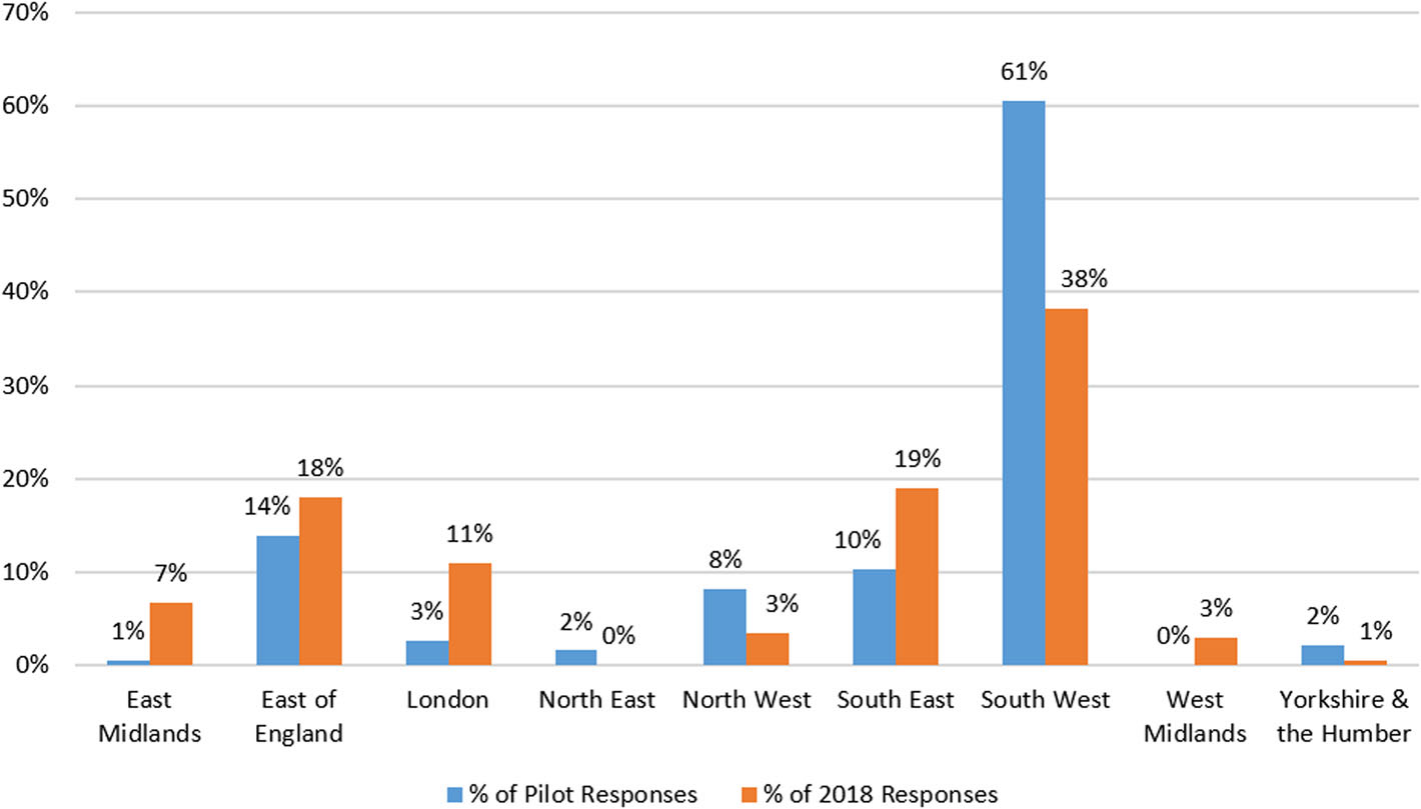
Percentage of total GP responses per English region and by survey

Use of regional organisations’ mailing lists was an effective tool for data collection but tended to result in a higher number of responses from that geographic area. During the pilot, the survey was emailed to GPs via the Clinical Research Network (CRN) South West, resulting in 200 responses. However, 61% of these came from the South West. In 2018, emails via a spread of English CRNs led to a 94% increase in GP responses (387) and also resulted in less dominance of responses from the South West (38% of responses, see Fig. 4).

**Fig. 5:**
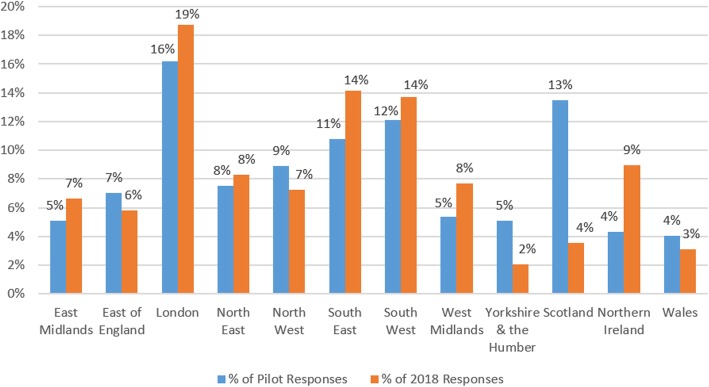
Percentage of psychiatrist responses by UK region and by survey

By contrast, emails distributed by organisations with a national reach, such as the Royal College of Psychiatrists, were associated with a relatively even spread of responses by geographic region (see Fig. 5).

#### Minimum responses

**Fig. 6:**
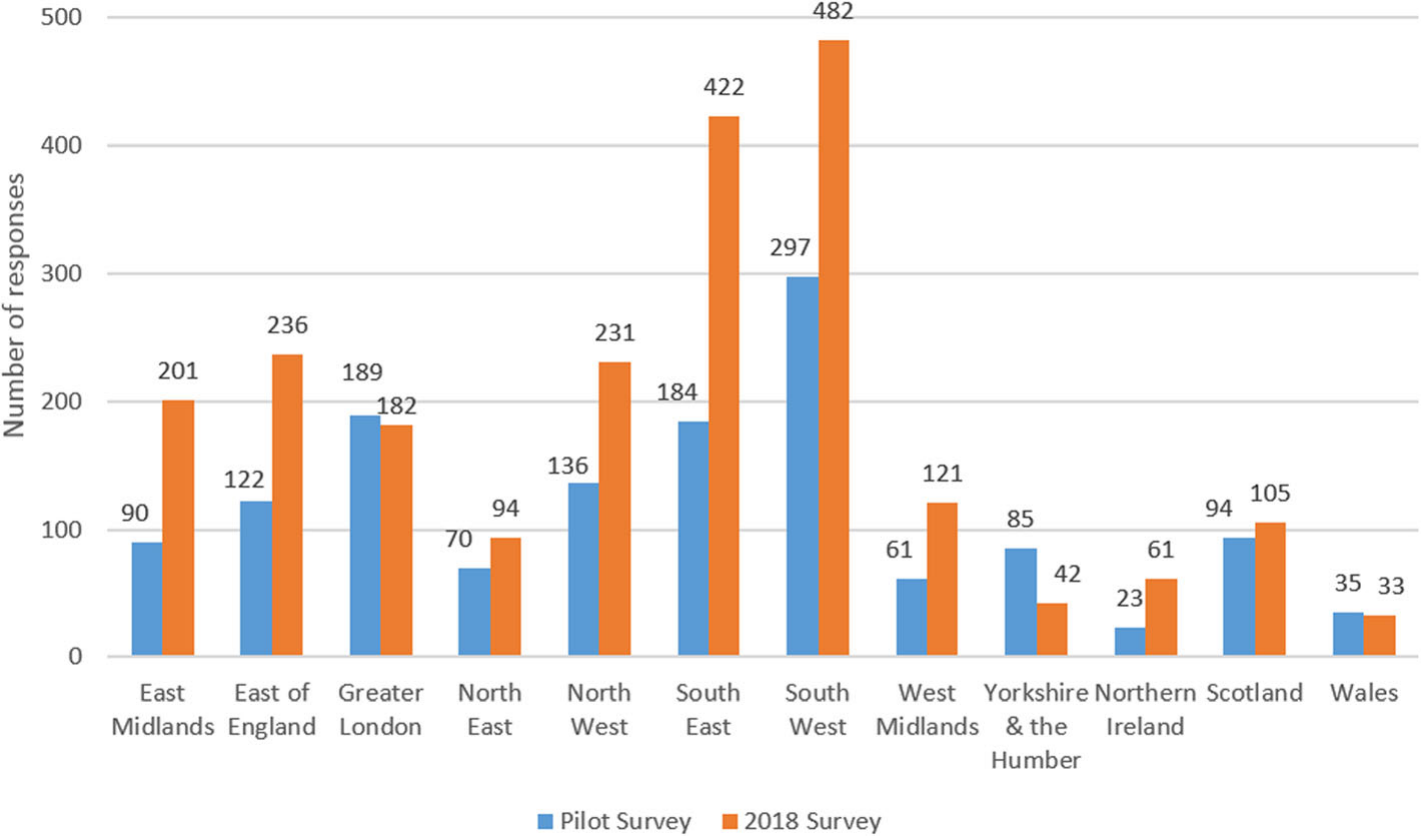
Number of responses by UK region and by survey

**Fig. 7:**
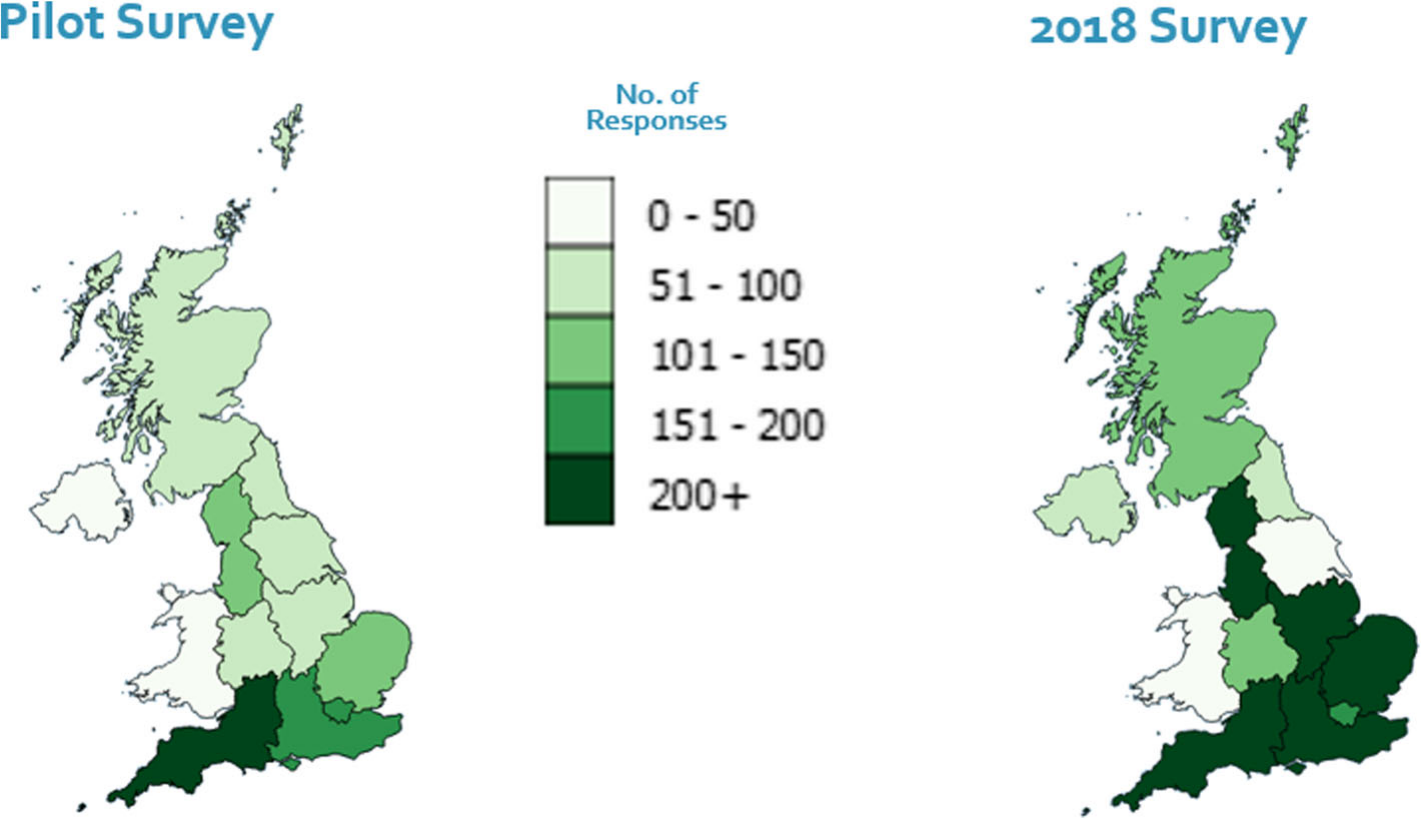
Response numbers by UK region for Pilot and 2018 surveys

Strategies aimed at increasing response numbers in 2018, were combined with targeting of under-represented regions and stakeholder groups part way through data collection, which led to an increase in regions with 51 or more informants (see Figs. 6 and 7). However, responses for Yorkshire and the Humber dropped from 85 to 42, and for Wales response numbers remained low at 33.

### Data analysis and handling

#### Data handling

Analysing pilot data was challenging. Data from multiple surveys required merging, and different questions by informant group made comparison difficult. Double data entry was used to reduce risks of data processing errors. Use of a single survey in 2018 made comparison of data provided by different informant groups’ straightforward and reduced the risk of data processing errors.

#### Identifying services

Service identification was faster in 2018 as the pre-populated list of services reduced the instances of services being identified by informants using free text (and thus needing hand matching to online information) from 100 to 16% of total instances (see Table 5).

**Table 5:** Numbers of unique services identified, and incidences of services being identified, by survey

Survey	Pilot	2018 Survey
Incidence^a^ of services being identified via free text	789	543
Incidence^a^ of services being identified via pre-populated list	–	3119
Total incidences^a^ of service identification	789	3662

^a^number of times any respondent indicated they knew of a service

#### Checking services

Narrowing the focus of service checking in 2018, combined with use of FOIs to NHS Trusts reduced the data processing burden. In the pilot, 83% of identified services (172 out of 208) met criteria of the second service definition, meaning they needed to be checked by researchers. Following 26 weeks spent contacting these services, 132 services had responded with data of mixed quality: a 77% response rate. By contrast, in 2018, only 23% of identified services (66 out of 292) met the narrower service checking criteria. These were checked via FOIs to 56 NHS trusts, with 49 responding within 10 weeks (an 88% response rate).

### Communicating findings

The QGIS software [30] allowed us to analyse the sample geographically and by stakeholder group, producing clear visual representations of response numbers by location (see Fig. 7).

Google My Maps, an interactive tool on which the service list could be uploaded for sharing, was an appropriate platform to communicate service locations. Presenting the final list of services (from the pilot) as a map, available via a research webpage, resulted in more than 34,000 views, indicating it was accessible and of interest to a large number of stakeholders [35].

### Hosting/updating service map

As services change and develop, service maps need active maintenance to remain accurate. The 2018 map was uploaded with clear information about when data collection took place [32]. A national ADHD professional body, that aims to support practitioners and provide information to all stakeholders, has agreed to host and update the map [33].

## Discussion

To expand and extend the findings from existing research [20, 24, 25], we piloted and refined methods for mapping adult ADHD services in the UK, with the aim of meeting current needs for national data about adult ADHD service provision. Adult ADHD continues to be under recognised in many countries, presenting particular barriers to caring for young people and adults with the condition [36], and mapping services can help to inform service development and reduce geographic health inequalities [16, 17, 21, 37]. We developed seven steps to map services rapidly, using available technology, and including the perspectives of a range of stakeholders.

Patient and public involvement (PPI) work with the parent advisory group, clinicians and commissioners, helped clarify the questions to ask. Research partnerships with clinical and ADHD support organisations made it possible to reach large numbers of informants across the UK and share findings. Piloting was instructive and important, as was a careful study of previous literature in order to produce a definitive map quickly and efficiently. Future research should aim to; gather background information on the range of services accessed by patients; develop a working definition of target services; and identify all key stakeholders, in advance of data collection.

A two-stage approach to the definition of the target service meant a wide range of services were indexed during data collection, with a narrower focus on NHS funded specialist ADHD services during analysis. The narrower focus on NHS funded specialist ADHD services, meant provision could be checked against government guidelines [12, 13, 38]. A balance needs to be struck between surveying all services relevant to stakeholders, which is time-consuming, but may reveal the sometimes hidden role of generic, voluntary and private services, and focussing on NHS specialist services. The first stage results in ‘messier’ data, but reflects stakeholders’ experiences of the complex nature of health service provision, providing validity.

Deciding on an appropriate way to narrow the focus when checking services was challenging due to high levels of heterogeneity in configuration of adult ADHD services in the UK [14], but the detailed pilot allowed us to make decisions about the narrower definitions that were based on empirical data. One recommended model of care, that of adult ADHD specialists working within general NHS mental health services [14], was not included in service checking, which is a limitation. However all generic NHS services identified by respondents as providing treatment/support were indexed. Limiting the 2018 map to specialist services for adults with ADHD funded by the NHS, produced a serviceable map in line with the study aims.

Surveying an inclusive range of informants served several functions. It helped reduce this risk of missing services [24] because multiple respondent types were asked about services in their area. It also allowed for comparisons between provider reports on service availability and patient experiences of provision. Findings are reported in full elsewhere [39]. Interestingly, over 40% of study informants identified themselves with two or more ADHD related roles, a reminder that survey respondents often occupy multiple identities [40]. Including of a range of informants is a strength when compared with mapping methods which rely on expert or government sources alone [18, 20]. However, the identification of key informants was more complex than expected; responses to the pilot, PPI work and previous literature all provided data that informed the final list.

During data collection, different dissemination methods appeared to work better for different stakeholders. Direct emails from trusted organisations gained high numbers of responses from busy clinicians. FOIs were effective when contacting commissioners, as they ensured someone with allocated time and resources received and responded to the query. FOIs can be a powerful tool for improving the transparency of mental health provision; which is known to be critical to delivering good outcomes and ensuring consistency of services [37, 41]. However care needs to be taken to ensure appropriate and responsible use, as FOIs have resource implications for the relevant public body [42]. Social media appeared to be a powerful and suitable resource for raising the profile of the survey [43]. Our use was mainly limited to Twitter, but this was an effective method of linking with ADHD focussed and appropriate clinical organisations and user groups. More proactive use of other social media formats might have increased response numbers from young adults with ADHD.

## Strengths and limitations

This methodology provides a blueprint for producing a definitive map quickly and efficiently. However, services are dynamic and service maps will need maintenance and clear information on the limitations of data accuracy. Although details of provision from dedicated services were checked, treatments provided by generic AMHS were not verified, meaning information was not gathered on all NHS services potentially treating adults with ADHD in line with NICE guidelines [12].

The aim of the survey was to gain a balance of responses, by geographic area and by stakeholder group, in order to achieve an accurate picture of service provision. This was achieved, although there were low response numbers from some regions and from some stakeholder groups, such as young adults with ADHD. A recent study looking at patterns of instant messaging use in students in the South West of England found 96% used Facebook and 59% used Instagram, compared with 58% using Twitter [44]. A more extensive use of social media formats that are accessible to young people with ADHD, such as Facebook (38), might have improved response numbers from these groups.

The online survey and data collection methods used were pragmatic and ‘fit for purpose’ [45], making use of technological advances to reach a wide audience rapidly and at relatively low cost. The non-probabilistic sampling methods allowed organisations to share the survey via their mailing lists without compromising data protection. It also facilitated link sharing across a variety of open forums. Respondents were not selected randomly and, except for commissioners, response rates could not be assessed, however this was not important as the aim was to gain an accurate and nuanced picture of service provision, not to generalise findings.

## Future work

Future mapping work should focus on checking details of provision offered by all adult NHS services that potentially meet NICE guidelines for treatment of adult ADHD [12]. Given sufficient resources, FOI requests sent to all provider organisations of AMHS could provide this information in a time efficient and cost effective way. However, a balance needs to be struck between additional research costs involved and benefits in terms of the quality of data received. This study and previous qualitative research has found a lack of clarity within generic AMHS on whether or not they provide treatment for adult ADHD [14, 26, 36]. Therefore it would be necessary to find a method of checking that services provide treatment in practice from the perspectives of a range of stakeholders, including service users.

Finding a way of sharing and updating the map in a way that is accessible to all stakeholders is important. It is a reflection of the quality of relationships built during the mapping exercise, and high levels of unmet need [36], that a national ADHD organisation has agreed to host the map [33]. The research team is supporting them to host this resource and update it into the future. This methodology could be usefully adopted to map health services for other conditions, such as ASD, where identified barriers to transition include a lack of comprehensive and integrated adult services [46, 47].

## Conclusion

This seven step process appears to be a pragmatic and efficient method for collating and communicating national service data about adult ADHD service provision in the UK. The inclusion of data from a range of stakeholders minimises the risk of missing information about services and allows comparison of perceptions of provision between commissioners, health workers and service users. We found information learned through PPI, and support from partner organisations both during the development of the surveys, data collection and for dissemination of results was crucial. Lessons learned here may be transferable to mapping service provision for other health conditions and in different contexts. For adult ADHD services, this method is an effective tool for quantifying provision and revealing gaps in service, so that, where indicated, an informed case can be made for change.

## Additional files

Additional file 1: Detailed Methodology. (DOCX 53 kb)

Additional file 2: 2018 Survey. (PDF 250 kb)

Additional file 3: 2016 Pilot Survey. (PDF 590 kb)

Additional file 4: Key Research Partners. (DOCX 12 kb)

Additional file 5: Checking Service Details. (DOCX 25 kb)

## Data Availability

The datasets generated and/or analysed during the current study are not publicly available because they are part of ongoing research that is not yet published, but they are available from the corresponding author on reasonable request.

## Abbreviations

ADHD: Attention-deficit/hyperactivity disorder
AMHS: Adult mental health services
ASD: Autism spectrum disorder
CAMHS: Child and adolescent mental health services
CATCh-uS: Children and Adolescents with ADHD in Transition between Child and Adult Services
CCGs: Clinical commissioning groups
CRN: Clinical Research Network
EU: European Union
FOI: Freedom of information requests
GPs: General practitioners
NHS: National Health Service
NIHR: National Institute for Health Research
QGIS: Open-source desktop geographic information system
UK: United Kingdom
