# Supplementary material for: Seven steps to mapping health service provision: lessons learned from mapping services for adults with Attention-Deficit/Hyperactivity Disorder (ADHD) in the UK
Source: BMC Health Serv Res. 2019 Jul 9;19:468. doi: 10.1186/s12913-019-4287-7 (PMC6617903; doi:10.1186/s12913-019-4287-7)
Supplement: Supplementary file 1 — Detailed Methodology. (DOCX 53 kb) [file 12913_2019_4287_MOESM1_ESM.docx]

**Detailed methodology**


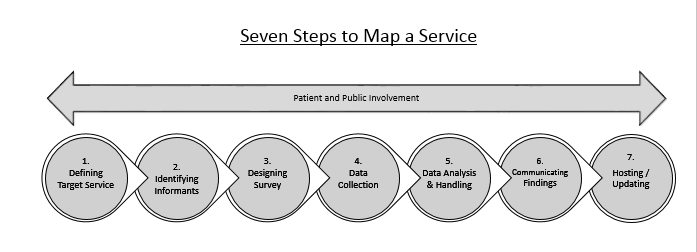
*Figure S2. Seven steps to mapping a health service*

**Patient and public involvement**

Patients and members of the public (including clinicians and commissioners) were involved with design, data collection and dissemination of research findings. We worked with an advisory group consisting of parents of young people with mental health difficulties including ADHD; consulting with them before the survey launch about who appropriate informants might be; checking language was accessible; testing early survey designs; requesting feedback on how to improve geographic spread and balance of responses; and sharing results from the pilot. Early survey designs were shared with representatives from each informant group and adjusted in line with feedback. Clinical and ADHD focussed organisations were contacted at various stages and asked to support distribution of the survey, communicate findings and to consider hosting/updating the service list after the research study finished.

**Step 1: Defining target service**

The UK National Institute for Health and Care Excellence guidelines state the following services should be available for adults with ADHD: transitional care, assessment and diagnostic services, drug titration, monitoring and review, and psychoeducation (1-3). The most recent guidance is that treatment should be holistic and provided by specialist teams with an expertise in ADHD. After titration and dose stabilisation, care may be carried out under shared care protocol arrangements with primary care (3). In addition, research indicates that due to gaps in services, adults with ADHD may either cease to access treatment or seek help at an extended range of services, including those not commissioned to treat adult ADHD (4).

*Pilot*: In light of the complex nature of the provision being mapped, two levels of service definition were employed. Firstly, a broad definition of “*any mental health service for people with ADHD aged 18 and above*” was used in the survey in order to record all services currently accessed by adults with ADHD. Secondly, during data analysis, the definition was narrowed so that only adult NHS specialist, private and charitable services with a focus on treating ADHD or neurodevelopmental conditions were checked to see if they supported/treated adults with ADHD. Applying a broad initial definition allowed us to later narrow the focus while remaining confident that we would be unlikely to miss any relevant services specialising in treating/supporting adults with ADHD.

*2018 Study:* Two levels of service definition were repeated and refined. The first-stage broad definition was kept, with notes added to the survey to make it explicit that a service could be a “*specialist doctor or team, mental health team, clinic, charity or support group that treats or supports adults with ADHD*”. At the second stage, in line with the aims of this research, the definition was narrowed to specialist adult ADHD services funded by the NHS. All services identified by informants were indexed, but only adult ADHD, ADHD and Autism Spectrum Disorder (ASD), Transition and Neurodevelopmental services were checked during data analysis to see if they offered services as recommended in the NICE guidelines (1-3).

**Step 2. Identifying informants**

The aim was to survey those receiving treatment/support (service users), those providing services (clinicians) and those funding services (commissioners). As NHS provision is underdeveloped and frequently seems not to adhere to NICE guidelines since 2008 (NICE), it was not immediately clear who those delivering care might be. Without a specialist service, treatment might be sought at a variety of services, including non-specialist NHS services, private and voluntary providers, and appropriate informants might provide treatment or funding for adults with ADHD at any of those services.

*Pilot*: Informants were initially identified by considering those in contact with young people with ADHD as they approach the age where they need to transition to adult services. This highlighted child and adolescent psychiatrists, adult psychiatrists and paediatricians, as well as young adults and their parents/carers. Clinical commissioning groups (CCGs) in England, Health Boards in Scotland and Wales and Health and Social Care Trusts in Northern Ireland are, to varying degrees, responsible for planning and commissioning health care services for their local area and therefore hold a key role in funding adult ADHD services. Different informants in different areas held varied knowledge and understanding of services, which emphasised the need to consult the widest range of stakeholders possible in order to be confident that all relevant services were identified. *2018 Survey*: In addition to informants identified for the pilot, general practitioners (GPs), nurses, practice managers and administrators were included, as it had become clear from pilot responses that individuals in these roles also played a significant part in service provision. Pilot responses revealed that many informants identified with more than one role. Therefore, respondents were given the opportunity to indicate additional roles. Respondents might, for example, indicate ‘*Parent of child with ADHD’* was their main role while also being a ‘*Psychiatrist’* and ‘*Adult with ADHD’*. Health commissioners across the UK were also identified.

**Step 3. Designing the survey**

An online survey method, Survey Monkey, was used as a pragmatic way of gathering data from a wide range of UK stakeholders, with the aim of covering a wide geographic area and minimising gaps in the data. Surveys were designed using lay terms to be accessible to all informants.

*Pilot*: The survey was made up of 9-15 questions and collected demographic and location specific information about respondents. Survey respondents were asked to identify themselves as a: young person, young adult, parent/carer, clinician or other. If the response was ‘other’, details could be provided using free text. Five core questions asked whether respondents knew of any services for adults with ADHD in their area, and if they did, to provide details. Wording and content were developed iteratively in consultation with parents/carers, clinicians and commissioners to ensure relevance and acceptability. Separate versions were created for each stakeholder group to enable collection of detailed demographics (see appendix B).

*2018 Survey*: The survey was brief, with 5-9 questions, and was merged into a single version for all respondents (see appendix C). Basic demographic information was collected and questions about informant location were limited to a predefined list of UK regions and postcodes to simplify data collection. The same core question was asked about knowledge of services, but without requesting treatment details as responses to these questions in the pilot had been varied and unreliable. Respondents were shown a list of services identified in the pilot and given the opportunity to indicate which of these they knew of, before being asked to provide details of any other services. After identifying a service, informants were asked to indicate if they or anyone they knew had experience of using that service for treatment/support of adult ADHD. This question meant services that were ‘known of’ could be separated from those that informants had ‘experience with’ in relation to adult ADHD support.

**Step 4. Data collection**

*Pilot*: Survey links were distributed to informants via three main methods: direct email from mailing lists of national organisations; in newsletters; and on websites. Awareness of the research was raised through university press releases, conference presentations and social media (Twitter). Initially, the survey was emailed to CCGs in England. Following review this strategy was changed to use of freedom of information (FOI) requests. FOIs give individuals the right to access recorded information held by public sector organisations (5).

*2018 Survey*: Data collection was planned in advance with research partners such as AADD-UK, the ADHD Foundation and the Royal Colleges of Psychiatrists (see appendix D). Where possible, emails were sent out via research partners’ mailing lists and used to share a link to the survey. The National Institute for Health Research (NIHR) funded Clinical Research Network (CRN) South West arranged to distribute emails via all regional CRNs, specifically targeting nurses, GPs, managers and clinical psychologists. The Twitter strategy focussed on sharing the survey link (possible because of the single survey design) and tagging appropriate organisations.

In preparation for the 2018 survey, a UK-wide dissemination strategy was planned, including contacting health boards in Scotland and Wales and trusts in Northern Ireland, as the pilot survey had not contacted funders/commissioners from these areas. Part-way through data collection, we checked responses to identify under-represented locations/informant groups. This allowed subsequent targeting of ADHD focussed and clinical organisations in those locations, with the aim of improving the balance of responses.

**Step 5. Data analysis and handling**

*Pilot*: Survey responses were uploaded into Excel, reviewed by two researchers and checked against the limited information available online to create a list of identified services. Those potentially offering support/treatment to adults with ADHD, were contacted by a research nurse via phone or email to confirm the type of service (for example, private, NHS, voluntary, specialist or generic) and details of treatments available. Child services were excluded from service checking.

*2018 Survey*: During data collection, response balance was assessed using Survey Monkey. Then data was uploaded into Excel and analysed using STATA SE15. Responses were checked against online information to create a list of identified services. Online information was often out of date or didn’t specify whether adult ADHD was treated, but checking allowed researchers to link identified services with the relevant organisational provider. Services potentially meeting the definition of dedicated adult ADHD services funded by the NHS were checked by sending FOI requests to the relevant health trust, to confirm details of provision (see appendix E). Child and adolescent, generic adult mental health, privately funded and voluntary services were excluded from service checking.

**Step 6. Communicating findings**

Response numbers were presented by informant group and location using a geographic information system, QGIS 2.18, to analyse and display the data (6). Shapefiles for UK counties and regions were imported (7, 8). Checked services, with details, were listed in Excel and uploaded to an interactive Google My Map.

*Pilot*: A Google map of checked services was posted onto the project website (9). The map included a disclaimer stating it was ‘*a work in progress…and not definitive’* (9). Services were categorised as NHS specialist, charity or private. Partner organisations embedded links to the map on their websites. Findings were shared via social media.

*2018 Survey*: Results were communicated using the same methods as for the pilot except specialist services were re-maned as ‘dedicated’ services to include ADHD clinics within generic AMHS. These are reported elsewhere in full (10).

**Step 7. Hosting/updating service map**

The pilot service map was updated in 2018, following the definitive survey. Two partner organisations, AADD-UK and UK Adult ADHD Network, were invited to host (and potentially update) the map of adult ADHD services once the research ends.

**References**

1. NICE. Attention deficit hyperactivity disorder: Diagnosis and management of ADHD in children, young people and adults (CG72) 2008 [cited 2017 3rd April]. Available from: <http://publications.nice.org.uk/attention-deficit-hyperactivity-disorder-cg72>.

2. NICE. Attention deficit hyperactivity disorder: Diagnosis and management of ADHD in children, young people and adults (CG72) 2016 [

3. NICE. Attention deficit hyperactivity disorder: diagnosis and management (NG87) 2018 [cited 2019 29th March]. Available from: <https://www.nice.org.uk/guidance/ng87/chapter/Recommendations>.

4. Wong IC, Asherson P, Bilbow A, Clifford S, Coghill D, DeSoysa R, et al. Cessation of attention deficit hyperactivity disorder drugs in the young (CADDY)--a pharmacoepidemiological and qualitative study. Health Technol Assess. 2009;13(50):iii-iv, ix-xi, 1-120.

5. Information Commissioners Office. The Guide to Freedom of Information 2016 [Available from: <http://www.legislation.gov.uk/ukpga/2000/36/contents>.

6. QGIS Development Team. QGIS Geographic information system, version 2.18. Open Source Geospatial Foundation Project. <http://qgis.osgeo.org2018>.

7. Office for National Statistics. Output Areas (OA) Boundaries 2016 [Available from: <https://data.gov.uk/dataset/0bf536b7-466f-4e6b-a515-751903972dcf/output-areas-oa-boundaries>.

8. McGarva G. English Government Office Network Regions (GOR) 2017 [Available from: <https://datashare.is.ed.ac.uk/handle/10283/2404>.

9. University of Exeter. CATCh-uS Mapping study - map of services 2018 [cited 2019 29th March]. Available from: <http://medicine.exeter.ac.uk/catchus/mapping/adhdservices/>.

10. Janssens A, Eke H, Price A, Newlove-Delgado T, Blake S, Ani C, et al. Young people with Attention Deficit Hyperactivity Disorder (ADHD) in transition from children's services to adult services (Catch-uS): a mixed methods project using national surveillance, qualitative and mapping studies Health Services and Delivery ResearchIn preparation [cited 2019 29th March]. Available from: <https://www.journalslibrary.nihr.ac.uk/programmes/hsdr/142152/#/>.
