# Supplementary material for: Seven steps to mapping health service provision: lessons learned from mapping services for adults with Attention-Deficit/Hyperactivity Disorder (ADHD) in the UK
Source: BMC Health Serv Res. 2019 Jul 9;19:468. doi: 10.1186/s12913-019-4287-7 (PMC6617903; doi:10.1186/s12913-019-4287-7)
Supplement: Supplementary file 2 — 2018 Survey. (PDF 250 kb) [file 12913_2019_4287_MOESM2_ESM.pdf]

2018 CATCh-uS: Children and adolescents with Attention Deficit Hyperactivity Disorder in transition between children's services and adult services

Mapping the availability of services for people aged 18 and above with ADHD

The CATCh-uS study is funded by the National Institute for Health Research and has ethical approval. Details can be found on our website. The aim of this survey is to map available services for people with Attention Deficit Hyperactivity Disorder (ADHD) who are aged 18 and over. This is to inform and improve service provision for young adults with ADHD. We only want to know about services you are aware of and/or have used for the management of ADHD. We will not share any of your personal information. The survey is anonymous and confidential. Answers will help to update a list of existing services, available here.

The survey should take no longer than 5 minutes to complete.

Example of website with service details...

* Are you...?
(Please choose whichever describes you best)

- ◯ Young person with ADHD (up to 17 years old)
- ◯ Adult with ADHD (aged 18+)
- ◯ Parent/carer/partner of someone with ADHD
- ◯ In an ADHD support role (e.g. voluntary, support work or training)
- ◯ Paediatrician
- ◯ Psychiatrist
- ◯ Clinical Psychologist
- ◯ Educational Practitioner (e.g. Support worker, Teacher, Behavioural Support, Ed Psych, EWO)
- ◯ Nurse
- ◯ GP
- ◯ Manager
- ◯ Allied Health Professional
- ◯ Researcher or Academic
- ◯ Administrator
- ◯ Clinical Commissioner
- ◯ Other (please specify)

Are you also...?
(If any of the options below also describe you, please tick all that apply)

- [ ] Young person with ADHD (up to 17 years old)
- [ ] Adult with ADHD (aged 18+)
- [ ] Parent/carer/partner of someone with ADHD
- [ ] In an ADHD support role (e.g. voluntary, support work or training)
- [ ] Paediatrician
- [ ] Psychiatrist
- [ ] Clinical Psychologist
- [ ] Educational Practitioner (e.g. Support worker, Teacher, Behavioural Support, Ed Psych, EWO)
- [ ] Nurse
- [ ] GP
- [ ] Manager
- [ ] Allied Health Professional
- [ ] Researcher or Academic
- [ ] Administrator
- [ ] Clinical Commissioner
- [ ] Other (please specify)

CATCh-uS

2018 CATCh-uS: Children and adolescents with Attention Deficit Hyperactivity Disorder in transition between children's services and adult services

Mapping the availability of services for people aged 18 and above with ADHD

If you work with people with ADHD please answer the following question (**if not, please skip**).
Which of the following sectors do you work in?
(Tick all that apply)

- [ ] Academia or Research
- [ ] Community Interest Company (or equivalent) providing for NHS
- [ ] NHS
- [ ] Private Provider for NHS
- [ ] Private Sector
- [ ] Voluntary Sector
- [ ] Education
- [ ] Other (please specify)

CATCh-uS

2018 CATCh-uS: Children and adolescents with Attention Deficit Hyperactivity Disorder in transition between children's services and adult services

Mapping the availability of services for people aged 18 and above with ADHD

* Do you know of any mental health services* (provided by NHS or other organisations) for people with ADHD aged 18+?

- ( ) Yes
- ( ) No

*A service could be NHS, private, a transition team, a charity or support group.

CATCh-uS

2018 CATCh-uS: Children and adolescents with Attention Deficit Hyperactivity Disorder in transition between children's services and adult services

Mapping the availability of services for people aged 18 and above with ADHD

UK regions...

We want to know who is responding to our survey, so please let us know where you live*. All information will be treated confidentially, with data stored securely and only used for the purpose of mapping areas responses come from.

* What is your postcode?
(For example EX1 2LU)

[ ]

* Which region of the UK are you based in?

[ ]

*If you are a clinician or working with people with ADHD, please choose the postcode and region of your workplace.

CATCh-uS

2018 CATCh-uS: Children and adolescents with Attention Deficit Hyperactivity Disorder in transition between children’s services and adult services

East Midlands & Eastern: Mapping availability of services for people aged 18 and above with ADHD

Here is a list of services that were identified in our 2016 survey. Please tick the services that you believe treat or support adults with ADHD.
(NOTE: not all of these have confirmed that they treat adult ADHD)

- [ ] ADD Norfolk; Support Group, NR7 9QL

- [ ] ADHD Foundation; National Charity, L2 2AH
- [ ] ADHD Solutions CIC; Charity/Voluntary, LE4 7GH
- [ ] Anchor Psychiatry Group; Private Provider, NR6 5BE
- [ ] Aspergers East Anglia; Aspergers Support Group, NR2 1DN
- [ ] Autism & ADHD Ipswich; Social Enterprise, IP2 8SJ
- [ ] Blackpool Teaching Hospitals NHS FT; 'Learning Disability', LA1 4JJ
- [ ] Cambridgeshire & Peterborough NHS FT; Adult Mental Health Service, PE29 3RJ
- [ ] Cambridgeshire & Peterborough NHS FT; Specialist Adult ADHD, Cambridge, CB21 5EF
- [ ] Cambridgeshire & Peterborough NHS FT; Specialist Adult ADHD, Peterborough, PE3 9GZ
- [ ] Derbyshire Healthcare NHS FT; 'Bolsover & Clay Cross Neighbourhood Team', Children's Service, S446HF
- [ ] Derbyshire Healthcare NHS FT; 'Learning Disability', DE22 3LZ
- [ ] Derbyshire Healthcare NHS FT; 'Mental Health & Emotional Wellbeing Service', Community Service, DE22 3LZ
- [ ] Hertfordshire Partnership University NHS FT; 'Learning Disability', NR13 5EW
- [ ] Leicestershire Partnership NHS Trust; Adult ADHD, LE4 8PQ
- [ ] Leicestershire Partnership NHS Trust; 'Adult Mental Health', LE3 0LP
- [ ] Lincolnshire Partnership NHS FT; 'Adult Community Mental Health Team', NG34 8GG
- [ ] MIND; National Charity, E15 4BQ
- [ ] NHS England; National Service 'Improving Access to Psychological Therapies (IAPT)', Mental Health Triage, LS2 7UE
- [ ] Norfolk & Suffolk NHS FT; Adult ADHD Service, NR6 5BE
- [ ] Norfolk & Suffolk NHS FT; 'Child, Family & YP Service', Central Norfolk, NR1 3RE
- [ ] Norfolk & Suffolk NHS FT; 0-25 Service, NR1 3RE
- [ ] Norfolk & Suffolk NHS FT; Community Mental Health Services, Great Yarmouth & Waveney, NR30 1BU
- [ ] Norfolk & Suffolk NHS FT; Integrated Delivery Team, (Bury South), Community Mental Health Service, NR6 5BE
- [ ] Norfolk & Suffolk NHS FT; Integrated Delivery Team, (Ipswich) Community Mental Health, IP1 2GA
- [ ] Norfolk & Suffolk NHS FT; 'Norfolk & Waveney Wellbeing', Mental Health Triage, NR6 5BE
- [ ] Norfolk & Suffolk NHS FT; West Norfolk, Community Mental Health Service, PE30 5PD
- [ ] Northern Lincolnshire & Goole NHS FT; Adult Mental Health Service, DN32 7DL
- [ ] Northern Lincolnshire & Goole NHS FT; Childrens Service, DN33 2BA
- [ ] Nottingham Adult ADHD; Support Group, NG1 5JT
- [ ] Nottingham University Hospitals NHS Trust; 'Beeston Health Clinic', Children's Service, NG9 2NR
- [ ] Nottinghamshire Healthcare NHS FT; 'Adult ADHD Clinic', NG7 2UH
- [ ] Nottinghamshire Healthcare NHS FT; 'CAMHS Developmental Neuropsychiatry & Tourette's Clinic', NG7 2UH
- [ ] Nottinghamshire Healthcare NHS FT; 'Community Mental Health Team for Adults', NG4 3AY

- [ ] Rotherham Doncaster & South Humber NHS FT; Adult Mental Health Service, DN16 2JX
- [ ] Rotherham Doncaster & South Humber NHS FT; 'CAMHS (St Nicholas House)', DN4 8QN
- [ ] Rotherham Doncaster & South Humber NHS FT; Doncaster, Specialist Adult ADHD, DN4 8QN
- [ ] Rotherham Doncaster & South Humber NHS FT; 'Rotherham Mental Health Hospital Liasion Team', S60 2UD
- [ ] Sheffield Children's NHS FT; 'Child Development & Neurodisability Service', S10 5DD
- [ ] Sheffield Health & Social Care NHS FT; 'Sheffield Autism & Neurodevelopment', S11 9BF
- [ ] South London & Maudsley NHS FT; National Adult ADHD Service, SE5 8AZ
- [ ] United Lincolnshire Hospitals NHS Trust; Children's Service, LN2 5QY

If none of the above, please click next.

CATCh-uS

2018 CATCh-uS: Children and adolescents with Attention Deficit Hyperactivity Disorder in transition between children’s services and adult services

Greater London: Mapping the availability of services for people aged 18 and above with ADHD

Here is a list of services that were identified in our 2016 survey. Please tick the services that you believe treat or support adults with ADHD.
(NOTE: not all of these have confirmed that they treat adult ADHD)

- [ ] ADDISS Support Service; Support Group, HA8 9HL
- [ ] ADHD Foundation; National Charity, L2 2AH
- [ ] Barnet, Enfield & Haringey MH NHS Trust; 'CAMHS', HA8 0AD
- [ ] Barnet, Enfield & Haringey MH NHS Trust; 'Enfield Assessment Service', Mental Health Triage, N14 5SH
- [ ] Barnet, Enfield & Haringey MH NHS Trust; Specialist Adult ADHD, HA5 3EE
- [ ] Camden & Islington NHS FT; 'Community Mental Health Team', NW1 0PE
- [ ] Camden & Islington NHS FT; 'Learning Disability', Camden, WC1H 9JE
- [ ] Camden & Islington NHS FT; 'Learning Disability', Islington, N5 1NS
- [ ] Central & NW London NHS FT; 'CAMHS', W8 7NX
- [ ] Central & NW London NHS FT; 'Recovery team East/West (Hounslow)', Community Mental Health Service, TW3 1SE
- [ ] Central & NW London NHS FT; Specialist Adult ADHD, HA1 3UJ

- [ ] Centre for ADHD & Autism Support; Support Group, HA4 9XA
- [ ] East London NHS FT; 'City & Hackney AMH Referral & Assessment', Mental Health Triage, E9 6SR
- [ ] East London NHS FT; 'Hackney Directory North Locality Team', Community Mental Health Service, N16 OLN
- [ ] Fast Minds; Support Group, KT1 2PT
- [ ] Guy's & St Thomas' NHS FT; 'Community Paediatrics', SE1 9RT
- [ ] Guy's & St Thomas' NHS FT; 'Mary Sheridan Centre for Child Health', Community Mental Health Service, SE11 4TH
- [ ] Guy's & St Thomas' NHS FT; 'TANDeM Team', Child Neurodevelopmental Service, SE1 7EH
- [ ] Homerton University Hospital NHS FT; 'CAMHS First Steps', E8 2FP
- [ ] Islington ADHD; Support Group, N1 2FB
- [ ] MIND; National Charity, E15 4BQ
- [ ] NHS England; National Service 'Improving Access to Psychological Therapies (IAPT)', Mental Health Triage, LS2 7UE
- [ ] Oxleas NHS FT; 'Locality Mental Health Service', Community Mental Health Service, DA2 7WG
- [ ] Private Practice; 'Professor Peter Hill; Psychiatrist', W1G 6AZ
- [ ] South London & Maudsley NHS FT; Lewisham, 'Autism & ADHD Service', SE13 6LW
- [ ] South London & Maudsley NHS FT; Croydon, Specialist Adult ADHD, CR0 2UR
- [ ] South London & Maudsley NHS FT; National Adult ADHD Service, SE5 8AZ
- [ ] Surrey & Borders Partnership NHS FT; 'Community Learning Disability', KT19 8PH
- [ ] Surrey & Borders Partnership NHS FT; 'Community Mental Health Recovery Service: Epsom', KT19 8PB
- [ ] SW London & St George's Mental Health NHS Trust; 'Merton Specialist Adult ADHD', CR4 4TP
- [ ] SW London & St George's Mental Health NHS Trust; 'Richmond Specialist Adult ADHD', TW9 2TE
- [ ] Unique Adult ADHD; Support Group, TW17 9DH
- [ ] Your Health Care; Richmond Adult ADHD Service, Community Interest Company, KT1 4DY

If none of the above, please click next.

CATCh-uS

2018 CATCh-uS: Children and adolescents with Attention Deficit Hyperactivity Disorder in transition between children’s services and adult services

North East: Mapping the availability of services for people aged 18 and above with ADHD

Here is a list of services that were identified in our 2016 survey. Please tick the services that you believe treat or support adults with ADHD.
(NOTE: not all of these have confirmed that they treat adult ADHD)

- ☐ AADD-UK; 'Adult ADHD North East', Support Group, NE3 3XT
- ☐ ADHD Foundation; National Charity, L2 2AH
- ☐ County Durham & Darlington NHS FT; 'Mental Health', Community Service, DL3 6HX
- ☐ Humber NHS FT; 'Community Mental Health', YO16 4ND
- ☐ Leeds & York Partnership NHS FT; Specialist Adult ADHD, LS2 9LN
- ☐ MIND; National Charity, E15 4BQ
- ☐ NHS England; National Service 'Improving Access to Psychological Therapies (IAPT)', Mental Health Triage, LS2 7UE
- ☐ Northumberland, Tyne & Wear NHS FT; 'Childrens & YP Service', NE21QE
- ☐ Northumberland, Tyne & Wear NHS FT; Specialist Adult ADHD, NE10 8DX
- ☐ South London & Maudsley NHS FT; National Adult ADHD Service, SE5 8AZ
- ☐ Spire Health Care, Private Provider, LS8 1NT
- ☐ SW Yorkshire Partnership NHS FT; 'Learning Disability', WF1 3SP
- ☐ SW Yorkshire Partnership NHS FT; Specialist Adult ADHD, WF1 5PN
- ☐ Tees, Esk & Wear Valleys NHS FT; 'Harrogate & Ripon Community Mental Health Service', HG1 2PW
- ☐ Tees, Esk & Wear Valleys NHS FT; Specialist Adult ADHD, TS18 3TS
- ☐ The Tower Clinic; 'Psychiatry', Private Provider, LS16 7AP
- ☐ The Tuke Centre; 'Autism & ADHD', Private Provider, YO10 5BN
- ☐ West Yorkshire ADHD; Support Group, WF1 4DG

If none of the above, please click next.

CATCh-uS

2018 CATCh-uS: Children and adolescents with Attention Deficit Hyperactivity Disorder in transition between children's services and adult services

North West: Mapping the availability of services for people aged 18 and above with ADHD

Here is a list of services that were identified in our 2016 survey. Please tick the services that you believe treat or support adults with ADHD.
(NOTE: not all of these have confirmed that they treat adult ADHD)

- [ ] 5 Boroughs Partnership NHS FT; Adult Mental Health Service, L35 5DR
- [ ] ADHD Foundation; National Charity, L2 2AH
- [ ] ADHD Lancashire Adult ADHD; Support Group, BB2 1NA
- [ ] Blackpool Teaching Hospitals NHS FT; Specialist Adult ADHD, FY1 6JX
- [ ] Bradford District NHS FT; 'CAMHS', BD7 1LN
- [ ] Cheshire & Wirral Partnership NHS FT; Wirral, Specialist Adult ADHD, CH42 0LQ
- [ ] Cumbria Partnership NHS FT; 'Community Mental Health Assessment & Recovery', CA11 8JA
- [ ] Greater Manchester West MH NHS FT; 'Manchester Lifeline ADHD', Specialist Adult ADHD, M25 3BL
- [ ] Greater Manchester West MH NHS FT; 'Tafford Extended Service', Adult Mental Health, M41 9HE
- [ ] Ladders of Life; 'Liverpool ADHD', Support Group, L5 3LA
- [ ] Lancashire Care NHS FT; 'Complex Care and Treatment Teams', Community Mental Health, PR5 6AW
- [ ] Lancashire Care NHS FT; Specialist Adult ADHD, BB5 5DE
- [ ] Mersey Care NHS FT; 'Aspergers & ADHD Service', PR9 0LT
- [ ] Mid Cheshire Hospitals NHS FT; 'Community Paediatrics', CW1 4QJ
- [ ] MIND; National Charity, E15 4BQ
- [ ] NHS England; National Service 'Improving Access to Psychological Therapies (IAPT)', Mental Health Triage, LS2 7UE
- [ ] North East Wales NHS Trust; 'Deeside Mental Health Resource Centre', Community Service, CH5 1XS
- [ ] Pennine Care NHS FT; 'CAMHS - Healthy Young Minds', OL6 7SR
- [ ] Pennine Care NHS FT; 'Oldham Learning Disability', OL4 1DE
- [ ] Priory Group; 'Mental Health ADHD', Private Provider, WA15 0NU
- [ ] Somerset Partnership NHS FT; 'Learning Disability', BA20 2BN
- [ ] South London & Maudsley NHS FT; National Adult ADHD Service, SE5 8AZ

If none of the above, please click next.

ADHD

Here is a list of services that were identified in our 2016 survey. Please tick the services that you believe treat or support adults with ADHD.
(NOTE: not all of these have confirmed that they treat adult ADHD)

- [ ] ADHD Foundation; National Charity, L2 2AH
- [ ] Belfast Health & Social Care Trust; 'CAMHS', BT97AB
- [ ] MIND; National Charity, E15 4BQ
- [ ] NHS England; National Service 'Improving Access to Psychological Therapies (IAPT)', Mental Health Triage, LS2 7UE
- [ ] Northern Health & Social Care Trust; 'CAMHS', BT41 2QB
- [ ] Northern Health & Social Care Trust; 'Community Mental Health', BT41 4AB
- [ ] Northern Health & Social Care Trust; Learning Disability, BT38 8AZ
- [ ] Northern Ireland & Southern Health & Social Care Trust; 'Outpatient Psychiatry Centre', BT61 7QG
- [ ] South Eastern Health & Social Care Trust; 'Adult services', BT28 1JP
- [ ] South London & Maudsley NHS FT; National Adult ADHD Service, SE5 8AZ
- [ ] Western Health & Social Care Trust; 'Primary Care Liaison Service', BT47 6SB
- [ ] Western Health & Social Care Trust; 'Recovery Mental Health Team', Adult Service, BT47 6AU

If none of the above, please click next.

CATCh-uS

2018 CATCh-uS: Children and adolescents with Attention Deficit Hyperactivity Disorder in transition between children's services and adult services

Scotland: Mapping the availability of services for people aged 18 and above with ADHD

Here is a list of services that were identified in our 2016 survey. Please tick the services that you believe treat or support adults with ADHD.
(NOTE: not all of these have confirmed that they treat adult ADHD)

- [ ] ADHD Foundation; National Charity, L2 2AH
- [ ] ADHD Parent Support West Glasgow; Support Group, G14 0BL
- [ ] MIND; National Charity, E15 4BQ
- [ ] NHS Borders; 'CAMHS', TD7 4LJ
- [ ] NHS Borders; 'East Community Mental Health Team', EH22 1BG
- [ ] NHS Borders; 'West Community Mental Health Team', TD1 3EB
- [ ] NHS Dumfries & Galloway, Adult Mental Health Service, DG1 4TG
- [ ] NHS England; National Service 'Improving Access to Psychological Therapies (IAPT)', Mental Health Triage, LS2 7UE
- [ ] NHS Fife, Adult Mental Health Service, KY2 5AH
- [ ] NHS Fife; 'Community Childrens ADHD Team', Child Neurodevelopmental Service, KY25AH
- [ ] NHS Grampian; 'Community Mental Health Teams', AB25 2ZH
- [ ] NHS Greater Glasgow & Clyde; 'Community Mental Health Team', G116HU
- [ ] NHS Greater Glasgow & Clyde; 'Community Mental Health Teams', PA16 0XN
- [ ] NHS Greater Glasgow & Clyde; 'West Centre Drumchapel', G15 7TS
- [ ] NHS Highlands; Adult Mental Health Services, Argyle and Bute Hospital', PA31 8LD
- [ ] NHS Highlands; 'Learning Disability', IV2 3DZ
- [ ] NHS Lanarkshire, Adult Mental Health Service, G71 8BB
- [ ] NHS Lanarkshire, East Kilbride CMH Trust; 'Special Interest Clinic, Outpatient Services - Hairmyres Hospital, G75 8RG
- [ ] NHS Lanarkshire; 'CAMHS', ML5 3AP
- [ ] NHS Lothian; 'Central Scotland ADHD', Specialist Adult ADHD, EH39 4QE
- [ ] NHS Lothian; 'Community Learning Disability Nurse', EH12 5AW
- [ ] NHS Lothian; 'Community Mental Health Service', EH105HF
- [ ] NHS Tayside; Adult Mental Health Service, DD1 9SY
- [ ] South London & Maudsley NHS FT; National Adult ADHD Service, SE5 8AZ

If none of the above, please click next.

CATCh-uS

South East: Mapping the availability of services for people aged 18 and above with ADHD

Here is a list of services that were identified in our 2016 survey. Please tick the services that you believe treat or support adults with ADHD.
(NOTE: not all of these have confirmed that they treat adult ADHD)

- ☐ ADHD Foundation; National Charity, L2 2AH
- ☐ Berkshire Healthcare NHS FT; Specialist Adult ADHD, RG1 5LR
- ☐ Change, Grow, Live; Tonbridge, Charity/Voluntary, TN9 1DH
- ☐ East London NHS FT; 'Adult Austism Service, Bedford', MK14 6AT
- ☐ East London NHS FT; 'Learning Disability', MK43 8HJ
- ☐ Hertfordshire Partnership University NHS FT; 'CAMHS', AL10 8YE
- ☐ Isle Of Wight NHS Trust Isle of Wight; Specialist Adult ADHD, PO37 7HZ
- ☐ Kent & Medway NHS & Social Care PT; 'ASD Diagnostic Service', ME16 9PH
- ☐ Kent Community Health NHS FT; 'Community Paediatrics', ME17 4AH
- ☐ Lanc UK; 'ADHD', Private Provider, RH12 2PD
- ☐ MIND; Brighton and Hove', Charity/Voluntary, BN2 3EW
- ☐ MIND; National Charity, E15 4BQ
- ☐ NHS England; National Service 'Improving Access to Psychological Therapies (IAPT)', Mental Health Triage, LS2 7UE
- ☐ North East London NHS FT; 'Community Mental health Service', AL108YE
- ☐ Oxford ADHD Centre; Private Provider, OX3 7BX
- ☐ Oxford Health NHS FT; 'Adult Mental Health Services', OX3 7JX
- ☐ Oxford Health NHS FT; 'Aylesbury Vale Adult Mental Health', HP20 1EG
- ☐ Oxford Health NHS FT; 'Community Mental Health Teams', HP13 6PT
- ☐ Pheonix Mental Health; Private Provider, HP10 0EU
- ☐ Psicon; 'Neurodevelopmental Lifespan', Private Provider, CT1 3AS
- ☐ South London & Maudsley NHS FT; National Adult ADHD Service, SE5 8AZ
- ☐ South London & Maudsley NHS FT; Specialist Adult ADHD, ME10 4XX
- ☐ Southern Health NHS FT; 'Community Learning Disability Team', OX16 2NT
- ☐ Sussex Partnership NHS FT; 'Learning Disability & Family Intensive Support Service', BN27 3DY
- ☐ Sussex Partnership NHS FT; 'Neurobehavioural Clinic', BN2 3EW
- ☐ Sussex Partnership NHS FT; Specialist Adult ADHD, BN2 3EW
- ☐ Sussex Partnership NHS FT; 'West Sussex Assessment & Treatment', Mental Health Triage, RH12 1RJ

If none of the above, please click next.

2018 CATCh-uS: Children and adolescents with Attention Deficit Hyperactivity Disorder in transition between children's services and adult services

South West: Mapping the availability of services for people aged 18 and above with ADHD

Here is a list of services that were identified in our 2016 survey. Please tick the services that you believe treat or support adults with ADHD.
(NOTE: not all of these have confirmed that they treat adult ADHD)

- [ ] 2gether NHS FT; 'Mental Health Liaison ', Specialist Adult ADHD, GL4 3GG
- [ ] AADD-UK; 'ADHD Bristol', Support Group, BS2 0EZ
- [ ] ADHD Foundation; National Charity, L2 2AH
- [ ] Avon & Wiltshire MH Partnership NHS Trust; Bristol Specialist Adult ADHD, BS14 9BP
- [ ] Avon & Wiltshire MH Partnership NHS Trust; 'CAMHS', SN15 1GG
- [ ] Avon & Wiltshire MH Partnership NHS Trust; 'Community Paediatrics, Westgate house', BS10 5NB
- [ ] Cornwall Partnership NHS FT; 'Community Mental Health Services', TR18 4NY
- [ ] Devon Partnership NHS Trust; 'Adult Mental Health', Community Mental Health Service, EX2 5AF
- [ ] Devon Partnership NHS Trust; 'Devon Autism & ADHD Service', EX2 7HU
- [ ] Devon Partnership NHS Trust; 'Devon Liaison, Diversion & Street Triage ', EX1 2LR
- [ ] Dorset Healthcare University NHS FT; 'Adult Mental Health Service', North and West Dorset, BH12 4NB
- [ ] Dorset Healthcare University NHS FT; 'CAMHS Dorset', BH17 0RB
- [ ] Dorset Healthcare University NHS FT; 'Community mental health (North & West Dorset)', BH17 0RB
- [ ] Dorset Healthcare University NHS FT; 'Community Paediatrics', BH17 0RB
- [ ] Dorset Healthcare University NHS FT; 'Learning Disability', BH17 0RB
- [ ] Dorset Healthcare University NHS FT; 'Steps to Wellbeing', Mental Health Triage, BH11 9TF
- [ ] Livewell Southwest; Plymouth 'Community Mental Health Team', Social Enterprise, PL4 7QD
- [ ] MIND; National Charity, E15 4BQ
- [ ] NHS England; National Service 'Improving Access to Psychological Therapies (IAPT)', Mental Health Triage, LS2 7UE
- [ ] North Devon Forum for ASD & ADHD; Support Group, EX39 5PX
- [ ] Somerset Partnership NHS FT; 'CAMHS', TA6 4RN
- [ ] Somerset Partnership NHS FT; 'Learning Disability', BA4 4PG
- [ ] Somerset Partnership NHS FT; 'Learning Disability', TA2 7PQ
- [ ] Somerset Partnership NHS FT; 'Learning Disability', TA6 5AT
- [ ] Somerset Partnership NHS FT; 'Older People's Community Mental Health', TA6 4RN
- [ ] Somerset Partnership NHS FT; 'Paediatric Service', TA6 4RN
- [ ] South London & Maudsley NHS FT; National Adult ADHD Service, SE5 8AZ
- [ ] Swindon ADHD; Support Group, SN1 2AU
- [ ] Taunton & Somerset NHS FT; 'Community Paediatrics', TA1 5DA
- [ ] Torbay & South Devon NHS FT; 'CAMHS', TQ7 2BA
- [ ] Virgin Care, Devon; 'CAMHS', EX2 4NU

If none of the above, please click next.

2018 CATCh-uS: Children and adolescents with Attention Deficit Hyperactivity Disorder in transition between children’s services and adult services

Wales: Mapping the availability of services for people aged 18 and above with ADHD

Here is a list of services that were identified in our 2016 survey. Please tick the services that you believe treat or support adults with ADHD.
(NOTE: not all of these have confirmed that they treat adult ADHD)

- [ ] Abertawe Bro Morgannwg University Health Board; 'Community Mental Health Team, West Glamorgan', Swansea, SA1 5AT
- [ ] ADHD Connections; Support Group, CF35 5HX
- [ ] ADHD Foundation; National Charity, L2 2AH
- [ ] Aneurin Bevan Health Board; 'Community Mental Health Teams', NP20 4PF
- [ ] Betsi Cadwaladr University Local Health Board; 'Mental Health Team/Psychiatry', LL18 5UJ
- [ ] Cardiff University; 'Child & Adolescent Psychiatry', CF14 7XB
- [ ] Cwm Taf Health Board; Adult Mental Health Service, CF72 8XR
- [ ] Cwm Taf Health Board; 'Royal Glamorgan Hospital Outpatient', Community Mental Health Service, CF72 8XR
- [ ] GIG Cymru NHS WALES; 'Ty Illtyd Mental Health Resource Centre', Community Mental Health Service, LD3 8AH
- [ ] MIND; National Charity, E15 4BQ
- [ ] NHS England; National Service 'Improving Access to Psychological Therapies (IAPT)', Mental Health Triage, LS2 7UE
- [ ] NHS Wales Gwynedd; 'South Gwynedd & Arfon Community Mental Health Team', LL40 1DU
- [ ] Powys Teaching Health Board; 'Bro Hafren Mental Health Resource Centre', Community Mental Health Service, SY16 2NG
- [ ] Powys Teaching Health Board; 'The Hazels Mental Health Resource Centre', Community Mental Health Service, LD1 5HF
- [ ] South London & Maudsley NHS FT; National Adult ADHD Service, SE5 8AZ

If none of the above, please click next.

West Midlands: Mapping the availability of services for people aged 18 and above with ADHD

Here is a list of services that were identified in our 2016 survey. Please tick the services that you believe treat or support adults with ADHD.
(NOTE: not all of these have confirmed that they treat adult ADHD)

- ☐ 2gether NHS FT; 'CAMHS', HR1 2HU
- ☐ ADHD Foundation; National Charity, L2 2AH
- ☐ ADHD Smart; Private Provider, NN4 8AF
- ☐ Birmingham & Solihull MH NHS FT; '0-25 Mental Health Service', B4 6DF
- ☐ Birmingham & Solihull MH NHS FT; 'Neuropsychiatry', B15 2FG
- ☐ Birmingham Community Healthcare NHS FT; 'Learning Disability', B13 8JL
- ☐ Coventry & Warwickshire Partnership NHS Trust; 'ADHD Transitions', CV2 2TE
- ☐ Coventry & Warwickshire Partnership NHS Trust; 'Community Mental Health Teams', B37 7HJ
- ☐ Dudley & Walsall MH Partnership NHS Trust; 'Neurodevelopmental', Dudley, DY1 2LZ
- ☐ Dudley & Walsall MH Partnership NHS Trust; 'Neurodevelopmental', Walsall, WS2 9XH
- ☐ F.A.C.T; 'Fact Northants', Support Group, NN3 2JF
- ☐ MIND; National Charity, E15 4BQ
- ☐ NHS England; National Service 'Improving Access to Psychological Therapies (IAPT)', Mental Health Triage, LS2 7UE
- ☐ North Staffordshire Combined Healthcare NHS Trust; 'Learning Disability', ST4 6TH
- ☐ Northamptonshire Healthcare NHS FT; 'ADHD & Aspergers Service', NN15 7PW
- ☐ South London & Maudsley NHS FT; National Adult ADHD Service, SE5 8AZ
- ☐ South Staffordshire & Shropshire Healthcare NHS FT; Adult Mental Health Service, ST16 3SR
- ☐ Sutton Medical Consulting Centre; Private Provider, B75 6DX

If none of the above, please click next.

CATCh-uS

East Midlands: Mapping the availability of services for people aged 18 and above with ADHD

These are the services you selected. Have you, or do you know someone aged 18+ who has received treatment or support at this service for their ADHD?
If yes, please tick.

- ☐ ADD Norfolk; Support Group, NR7 9QL
- ☐ ADHD Foundation; National Charity, L2 2AH
- ☐ ADHD Solutions CIC; Charity/Voluntary, LE4 7GH
- ☐ Anchor Psychiatry Group; Private Provider, NR6 5BE
- ☐ Aspergers East Anglia; Aspergers Support Group, NR2 1DN
- ☐ Autism & ADHD Ipswich; Social Enterprise, IP2 8SJ
- ☐ Blackpool Teaching Hospitals NHS FT; 'Learning Disability', LA1 4JJ
- ☐ Cambridgeshire & Peterborough NHS FT; Adult Mental Health Service, PE29 3RJ
- ☐ Cambridgeshire & Peterborough NHS FT; Specialist Adult ADHD, Cambridge, CB21 5EF
- ☐ Cambridgeshire & Peterborough NHS FT; Specialist Adult ADHD, Peterborough, PE3 9GZ
- ☐ Derbyshire Healthcare NHS FT; 'Bolsover & Clay Cross Neighbourhood Team', Children's Service, S446HF
- ☐ Derbyshire Healthcare NHS FT; 'Learning Disability', DE22 3LZ
- ☐ Derbyshire Healthcare NHS FT; 'Mental Health & Emotional Wellbeing Service', Community Service, DE22 3LZ
- ☐ Hertfordshire Partnership University NHS FT; 'Learning Disability', NR13 5EW
- ☐ Leicestershire Partnership NHS Trust; Adult ADHD, LE4 8PQ
- ☐ Leicestershire Partnership NHS Trust; 'Adult Mental Health', LE3 0LP
- ☐ Lincolnshire Partnership NHS FT; 'Adult Community Mental Health Team', NG34 8GG
- ☐ MIND; National Charity, E15 4BQ
- ☐ NHS England; National Service 'Improving Access to Psychological Therapies (IAPT)', Mental Health Triage, LS2 7UE
- ☐ Norfolk & Suffolk NHS FT; Adult ADHD Service, NR6 5BE
- ☐ Norfolk & Suffolk NHS FT; 'Child, Family & YP Service', Central Norfolk, NR1 3RE
- ☐ Norfolk & Suffolk NHS FT; 0-25 Service, NR1 3RE
- ☐ Norfolk & Suffolk NHS FT; Community Mental Health Services, Great Yarmouth & Waveney, NR30 1BU
- ☐ Norfolk & Suffolk NHS FT; Integrated Delivery Team, (Bury South), Community Mental Health Service, NR6 5BE
- ☐ Norfolk & Suffolk NHS FT; Integrated Delivery Team, (Ipswich) Community Mental Health, IP1 2GA
- ☐ Norfolk & Suffolk NHS FT; 'Norfolk & Waveney Wellbeing', Mental Health Triage, NR6 5BE
- ☐ Norfolk & Suffolk NHS FT; West Norfolk, Community Mental Health Service, PE30 5PD

- [ ] Northern Lincolnshire & Goole NHS FT; Adult Mental Health Service, DN32 7DL
- [ ] Northern Lincolnshire & Goole NHS FT; Childrens Service, DN33 2BA
- [ ] Nottingham Adult ADHD; Support Group, NG1 5JT
- [ ] Nottingham University Hospitals NHS Trust; 'Beeston Health Clinic', Children's Service, NG9 2NR
- [ ] Nottinghamshire Healthcare NHS FT; 'Adult ADHD Clinic', NG7 2UH
- [ ] Nottinghamshire Healthcare NHS FT; 'CAMHS Developmental Neuropsychiatry & Tourette's Clinic', NG7 2UH
- [ ] Nottinghamshire Healthcare NHS FT; 'Community Mental Health Team for Adults', NG4 3AY
- [ ] Rotherham Doncaster & South Humber NHS FT; Adult Mental Health Service, DN16 2JX
- [ ] Rotherham Doncaster & South Humber NHS FT; 'CAMHS (St Nicholas House)', DN4 8QN
- [ ] Rotherham Doncaster & South Humber NHS FT; Doncaster, Specialist Adult ADHD, DN4 8QN
- [ ] Rotherham Doncaster & South Humber NHS FT; 'Rotherham Mental Health Hospital Liasion Team', S60 2UD
- [ ] Sheffield Children's NHS FT; 'Child Development & Neurodisability Service', S10 5DD
- [ ] Sheffield Health & Social Care NHS FT; 'Sheffield Autism & Neurodevelopment', S11 9BF
- [ ] South London & Maudsley NHS FT; National Adult ADHD Service, SE5 8AZ
- [ ] United Lincolnshire Hospitals NHS Trust; Children's Service, LN2 5QY

CATCh-uS

2018 CATCh-uS: Children and adolescents with Attention Deficit Hyperactivity Disorder in transition between children's services and adult services

Greater London: Mapping the availability of services for people aged 18 and above with ADHD

These are the services you selected. Have you, or do you know someone aged 18+ who has received treatment or support at this service for their ADHD?
If yes, please tick.

- [ ] ADDISS Support Service; Support Group, HA8 9HL
- [ ] ADHD Foundation; National Charity, L2 2AH
- [ ] Barnet, Enfield & Haringey MH NHS Trust; 'CAMHS', HA8 0AD
- [ ] Barnet, Enfield & Haringey MH NHS Trust; 'Enfield Assessment Service', Mental Health Triage, N14 5SH
- [ ] Barnet, Enfield & Haringey MH NHS Trust; Specialist Adult ADHD, HA5 3EE

- [ ] Camden & Islington NHS FT; 'Community Mental Health Team', NW1 0PE
- [ ] Camden & Islington NHS FT; 'Learning Disability', Camden, WC1H 9JE
- [ ] Camden & Islington NHS FT; 'Learning Disability', Islington, N5 1NS
- [ ] Central & NW London NHS FT; 'CAMHS', W8 7NX
- [ ] Central & NW London NHS FT; 'Recovery team East/West (Hounslow)', Community Mental Health Service, TW3 1SE
- [ ] Central & NW London NHS FT; Specialist Adult ADHD, HA1 3UJ
- [ ] Centre for ADHD & Autism Support; Support Group, HA4 9XA
- [ ] East London NHS FT; 'City & Hackney AMH Referral & Assessment', Mental Health Triage, E9 6SR
- [ ] East London NHS FT; 'Hackney Directory North Locality Team', Community Mental Health Service, N16 OLN
- [ ] Fast Minds; Support Group, KT1 2PT
- [ ] Guy's & St Thomas' NHS FT; 'Community Paediatrics', SE1 9RT
- [ ] Guy's & St Thomas' NHS FT; 'Mary Sheridan Centre for Child Health', Community Mental Health Service, SE11 4TH
- [ ] Guy's & St Thomas' NHS FT; 'TANDeM Team', Child Neurodevelopmental Service, SE1 7EH
- [ ] Homerton University Hospital NHS FT; 'CAMHS First Steps', E8 2FP
- [ ] Islington ADHD; Support Group, N1 2FB
- [ ] MIND; National Charity, E15 4BQ
- [ ] NHS England; National Service 'Improving Access to Psychological Therapies (IAPT)', Mental Health Triage, LS2 7UE
- [ ] Oxleas NHS FT; 'Locality Mental Health Service', Community Mental Health Service, DA2 7WG
- [ ] Private Practice; 'Professor Peter Hill; Psychiatrist', W1G 6AZ
- [ ] South London & Maudsley NHS FT; Lewisham, 'Autism & ADHD Service', SE13 6LW
- [ ] South London & Maudsley NHS FT; Croydon, Specialist Adult ADHD, CR0 2UR
- [ ] South London & Maudsley NHS FT; National Adult ADHD Service, SE5 8AZ
- [ ] Surrey & Borders Partnership NHS FT; 'Community Learning Disability', KT19 8PH
- [ ] Surrey & Borders Partnership NHS FT; 'Community Mental Health Recovery Service: Epsom', KT19 8PB
- [ ] SW London & St George's Mental Health NHS Trust; 'Merton Specialist Adult ADHD', CR4 4TP
- [ ] SW London & St George's Mental Health NHS Trust; 'Richmond Specialist Adult ADHD', TW9 2TE
- [ ] Unique Adult ADHD; Support Group, TW17 9DH
- [ ] Your Health Care; Richmond Adult ADHD Service, Community Interest Company, KT1 4DY

CATCh-uS

2018 CATCh-uS: Children and adolescents with Attention Deficit Hyperactivity Disorder in transition between children’s services and adult services

North East: Mapping the availability of services for people aged 18 and above with ADHD

These are the services you selected. Have you, or do you know someone aged 18+ who has received treatment or support at this service for their ADHD?
If yes, please tick.

- ☐ AADD-UK; 'Adult ADHD North East', Support Group, NE3 3XT
- ☐ County Durham & Darlington NHS FT; 'Mental Health', Community Service, DL3 6HX
- ☐ ADHD Foundation; National Charity, L2 2AH
- ☐ Humber NHS FT; 'Community Mental Health', YO16 4ND
- ☐ Leeds & York Partnership NHS FT; Specialist Adult ADHD, LS2 9LN
- ☐ NHS England; National Service 'Improving Access to Psychological Therapies (IAPT)', Mental Health Triage, LS2 7UE
- ☐ Northumberland, Tyne & Wear NHS FT; 'Childrens & YP Service', NE21QE
- ☐ Northumberland, Tyne & Wear NHS FT; Specialist Adult ADHD, NE10 8DX
- ☐ MIND; National Charity, E15 4BQ
- ☐ Spire Health Care, Private Provider, LS8 1NT
- ☐ SW Yorkshire Partnership NHS FT; 'Learning Disability', WF1 3SP
- ☐ SW Yorkshire Partnership NHS FT; Specialist Adult ADHD, WF1 5PN
- ☐ Tees, Esk & Wear Valleys NHS FT; 'Harrogate & Ripon Community Mental Health Service', HG1 2PW
- ☐ Tees, Esk & Wear Valleys NHS FT; Specialist Adult ADHD, TS18 3TS
- ☐ South London & Maudsley NHS FT; National Adult ADHD Service, SE5 8AZ
- ☐ The Tower Clinic; 'Psychiatry', Private Provider, LS16 7AP
- ☐ The Tuke Centre; 'Autism & ADHD', Private Provider, YO10 5BN
- ☐ West Yorkshire ADHD; Support Group, WF1 4DG

CATCh-uS

2018 CATCh-uS: Children and adolescents with Attention Deficit Hyperactivity Disorder in transition between children’s services and adult services

North West: Mapping the availability of services for people aged 18 and above with ADHD

These are the services you selected. Have you, or do you know someone aged 18+ who has received treatment or support at this service for their ADHD?
If yes, please tick.

- ☐ 5 Boroughs Partnership NHS FT; Adult Mental Health Service, L35 5DR
- ☐ ADHD Foundation; National Charity, L2 2AH
- ☐ ADHD Lancashire Adult ADHD; Support Group, BB2 1NA
- ☐ Blackpool Teaching Hospitals NHS FT; Specialist Adult ADHD, FY1 6JX
- ☐ Bradford District NHS FT; 'CAMHS', BD7 1LN
- ☐ Cheshire & Wirral Partnership NHS FT; Wirral, Specialist Adult ADHD, CH42 0LQ
- ☐ Cumbria Partnership NHS FT; 'Community Mental Health Assessment & Recovery', CA11 8JA
- ☐ Greater Manchester West MH NHS FT; 'Manchester Lifeline ADHD', Specialist Adult ADHD, M25 3BL
- ☐ Greater Manchester West MH NHS FT; 'Tafford Extended Service', Adult Mental Health, M41 9HE
- ☐ Ladders of Life; 'Liverpool ADHD', Support Group, L5 3LA
- ☐ Lancashire Care NHS FT; 'Complex Care and Treatment Teams', Community Mental Health, PR5 6AW
- ☐ Lancashire Care NHS FT; Specialist Adult ADHD, BB5 5DE
- ☐ Mersey Care NHS FT; 'Aspergers & ADHD Service', PR9 0LT
- ☐ Mid Cheshire Hospitals NHS FT; 'Community Paediatrics', CW1 4QJ
- ☐ MIND; National Charity, E15 4BQ
- ☐ NHS England; National Service 'Improving Access to Psychological Therapies (IAPT)', Mental Health Triage, LS2 7UE
- ☐ North East Wales NHS Trust; 'Deeside Mental Health Resource Centre', Community Service, CH5 1XS
- ☐ Pennine Care NHS FT; 'CAMHS - Healthy Young Minds', OL6 7SR
- ☐ Pennine Care NHS FT; 'Oldham Learning Disability', OL4 1DE
- ☐ Priory Group; 'Mental Health ADHD', Private Provider, WA15 0NU
- ☐ Somerset Partnership NHS FT; 'Learning Disability', BA20 2BN
- ☐ South London & Maudsley NHS FT; National Adult ADHD Service, SE5 8AZ

ADHD

These are the services you selected. Have you, or do you know someone aged 18+ who has received treatment or support at this service for their ADHD?
If yes, please tick.

- [ ] ADHD Foundation; National Charity, L2 2AH
- [ ] Belfast Health & Social Care Trust; 'CAMHS', BT97AB
- [ ] MIND; National Charity, E15 4BQ
- [ ] NHS England; National Service 'Improving Access to Psychological Therapies (IAPT)', Mental Health Triage, LS2 7UE
- [ ] Northern Health & Social Care Trust; 'CAMHS', BT41 2QB
- [ ] Northern Health & Social Care Trust; 'Community Mental Health', BT41 4AB
- [ ] Northern Health & Social Care Trust; Learning Disability, BT38 8AZ
- [ ] Northern Ireland & Southern Health & Social Care Trust; 'Outpatient Psychiatry Centre', BT61 7QG
- [ ] South Eastern Health & Social Care Trust; 'Adult services', BT28 1JP
- [ ] South London & Maudsley NHS FT; National Adult ADHD Service, SE5 8AZ
- [ ] Western Health & Social Care Trust; 'Primary Care Liaison Service', BT47 6SB
- [ ] Western Health & Social Care Trust; 'Recovery Mental Health Team', Adult Service, BT47 6AU

CATCh-uS

2018 CATCh-uS: Children and adolescents with Attention Deficit Hyperactivity Disorder in transition between children’s services and adult services

Scotland: Mapping the availability of services for people aged 18 and above with ADHD

These are the services you selected. Have you, or do you know someone aged 18+ who has received treatment or support at this service for their ADHD?
If yes, please tick.

- ☐ ADHD Foundation; National Charity, L2 2AH
- ☐ ADHD Parent Support West Glasgow; Support Group, G14 0BL
- ☐ MIND; National Charity, E15 4BQ
- ☐ NHS Borders; 'CAMHS', TD7 4LJ
- ☐ NHS Borders; 'East Community Mental Health Team', EH22 1BG
- ☐ NHS Borders; 'West Community Mental Health Team', TD1 3EB
- ☐ NHS Dumfries & Galloway, Adult Mental Health Service, DG1 4TG
- ☐ NHS England; National Service 'Improving Access to Psychological Therapies (IAPT)', Mental Health Triage, LS2 7UE
- ☐ NHS Fife, Adult Mental Health Service, KY2 5AH
- ☐ NHS Fife; 'Community Childrens ADHD Team', Child Neurodevelopmental Service, KY25AH
- ☐ NHS Grampian; 'Community Mental Health Teams', AB25 2ZH
- ☐ NHS Greater Glasgow & Clyde; 'Community Mental Health Team', G116HU
- ☐ NHS Greater Glasgow & Clyde; 'Community Mental Health Teams', PA16 0XN
- ☐ NHS Greater Glasgow & Clyde; 'West Centre Drumchapel', G15 7TS
- ☐ NHS Highlands; Adult Mental Health Services, Argyle and Bute Hospital', PA31 8LD
- ☐ NHS Highlands; 'Learning Disability', IV2 3DZ
- ☐ NHS Lanarkshire, Adult Mental Health Service, G71 8BB
- ☐ NHS Lanarkshire, East Kilbride CMH Trust; 'Special Interest Clinic, Outpatient Services - Hairmyres Hospital, G75 8RG
- ☐ NHS Lanarkshire; 'CAMHS', ML5 3AP
- ☐ NHS Lothian; 'Central Scotland ADHD', Specialist Adult ADHD, EH39 4QE
- ☐ NHS Lothian; 'Community Learning Disability Nurse', EH12 5AW
- ☐ NHS Lothian; 'Community Mental Health Service', EH105HF
- ☐ NHS Tayside; Adult Mental Health Service, DD1 9SY
- ☐ South London & Maudsley NHS FT; National Adult ADHD Service, SE5 8AZ

CATCh-uS

South East: Mapping the availability of services for people aged 18 and above with ADHD

These are the services you selected. Have you, or do you know someone aged 18+ who has received treatment or support at this service for their ADHD?
If yes, please tick.

- ☐ ADHD Foundation; National Charity, L2 2AH
- ☐ Berkshire Healthcare NHS FT; Specialist Adult ADHD, RG1 5LR
- ☐ Change, Grow, Live; Tonbridge, Charity/Voluntary, TN9 1DH
- ☐ East London NHS FT; 'Adult Austism Service, Bedford', MK14 6AT
- ☐ East London NHS FT; 'Learning Disability', MK43 8HJ
- ☐ Hertfordshire Partnership University NHS FT; 'CAMHS', AL10 8YE
- ☐ Isle Of Wight NHS Trust Isle of Wight; Specialist Adult ADHD, PO37 7HZ
- ☐ Kent & Medway NHS & Social Care PT; 'ASD Diagnostic Service', ME16 9PH
- ☐ Kent Community Health NHS FT; 'Community Paediatrics', ME17 4AH
- ☐ Lanc UK; 'ADHD', Private Provider, RH12 2PD
- ☐ MIND; Brighton and Hove', Charity/Voluntary, BN2 3EW
- ☐ MIND; National Charity, E15 4BQ
- ☐ NHS England; National Service 'Improving Access to Psychological Therapies (IAPT)', Mental Health Triage, LS2 7UE
- ☐ North East London NHS FT; 'Community Mental health Service', AL108YE
- ☐ Oxford ADHD Centre; Private Provider, OX3 7BX
- ☐ Oxford Health NHS FT; 'Adult Mental Health Services', OX3 7JX
- ☐ Oxford Health NHS FT; 'Aylesbury Vale Adult Mental Health', HP20 1EG
- ☐ Oxford Health NHS FT; 'Community Mental Health Teams', HP13 6PT
- ☐ Pheonix Mental Health; Private Provider, HP10 0EU
- ☐ Psicon; 'Neurodevelopmental Lifespan', Private Provider, CT1 3AS
- ☐ South London & Maudsley NHS FT; National Adult ADHD Service, SE5 8AZ
- ☐ South London & Maudsley NHS FT; Specialist Adult ADHD, ME10 4XX
- ☐ Southern Health NHS FT; 'Community Learning Disability Team', OX16 2NT
- ☐ Sussex Partnership NHS FT; 'Learning Disability & Family Intensive Support Service', BN27 3DY
- ☐ Sussex Partnership NHS FT; 'Neurobehavioural Clinic', BN2 3EW
- ☐ Sussex Partnership NHS FT; Specialist Adult ADHD, BN2 3EW
- ☐ Sussex Partnership NHS FT; 'West Sussex Assessment & Treatment', Mental Health Triage, RH12 1RJ

2018 CATCh-uS: Children and adolescents with Attention Deficit Hyperactivity Disorder in transition between children's services and adult services

South West: Mapping the availability of services for people aged 18 and above with ADHD

These are the services you selected. Have you, or do you know someone aged 18+ who has received treatment or support at this service for their ADHD?
If yes, please tick.

- [ ] 2gether NHS FT; 'Mental Health Liaison ', Specialist Adult ADHD, GL4 3GG
- [ ] AADD-UK; 'ADHD Bristol', Support Group, BS2 0EZ
- [ ] ADHD Foundation; National Charity, L2 2AH
- [ ] Avon & Wiltshire MH Partnership NHS Trust; Bristol Specialist Adult ADHD, BS14 9BP
- [ ] Avon & Wiltshire MH Partnership NHS Trust; 'CAMHS', SN15 1GG
- [ ] Avon & Wiltshire MH Partnership NHS Trust; 'Community Paediatrics, Westgate house', BS10 5NB
- [ ] Cornwall Partnership NHS FT; 'Community Mental Health Services', TR18 4NY
- [ ] Devon Partnership NHS Trust; 'Adult Mental Health', Community Mental Health Service, EX2 5AF
- [ ] Devon Partnership NHS Trust; 'Devon Autism & ADHD Service', EX2 7HU
- [ ] Devon Partnership NHS Trust; 'Devon Liaison, Diversion & Street Triage ', EX1 2LR
- [ ] Dorset Healthcare University NHS FT; 'Adult Mental Health Service', North and West Dorset, BH12 4NB
- [ ] Dorset Healthcare University NHS FT; 'CAMHS Dorset', BH17 0RB
- [ ] Dorset Healthcare University NHS FT; 'Community mental health (North & West Dorset)', BH17 0RB
- [ ] Dorset Healthcare University NHS FT; 'Community Paediatrics', BH17 0RB
- [ ] Dorset Healthcare University NHS FT; 'Learning Disability', BH17 0RB
- [ ] Dorset Healthcare University NHS FT; 'Steps to Wellbeing', Mental Health Triage, BH11 9TF
- [ ] Livewell Southwest; Plymouth 'Community Mental Health Team', Social Enterprise, PL4 7QD
- [ ] MIND; National Charity, E15 4BQ
- [ ] NHS England; National Service 'Improving Access to Psychological Therapies (IAPT)', Mental Health Triage, LS2 7UE
- [ ] North Devon Forum for ASD & ADHD; Support Group, EX39 5PX
- [ ] Somerset Partnership NHS FT; 'CAMHS', TA6 4RN
- [ ] Somerset Partnership NHS FT; 'Learning Disability', BA4 4PG
- [ ] Somerset Partnership NHS FT; 'Learning Disability', TA2 7PQ
- [ ] Somerset Partnership NHS FT; 'Learning Disability', TA6 5AT
- [ ] Somerset Partnership NHS FT; 'Older People's Community Mental Health', TA6 4RN
- [ ] Somerset Partnership NHS FT; 'Paediatric Service', TA6 4RN
- [ ] South London & Maudsley NHS FT; National Adult ADHD Service, SE5 8AZ
- [ ] Swindon ADHD; Support Group, SN1 2AU
- [ ] Taunton & Somerset NHS FT; 'Community Paediatrics', TA1 5DA
- [ ] Torbay & South Devon NHS FT; 'CAMHS', TQ7 2BA
- [ ] Virgin Care, Devon; 'CAMHS', EX2 4NU

2018 CATCh-uS: Children and adolescents with Attention Deficit Hyperactivity Disorder in transition between children’s services and adult services

Wales: Mapping the availability of services for people aged 18 and above with ADHD

These are the services you selected. Have you, or do you know someone aged 18+ who has received treatment or support at this service for their ADHD?
If yes, please tick.

- [ ] Abertawe Bro Morgannwg University Health Board; 'Community Mental Health Team, West Glamorgan', Swansea, SA1 5AT
- [ ] ADHD Connections; Support Group, CF35 5HX
- [ ] ADHD Foundation; National Charity, L2 2AH
- [ ] Aneurin Bevan Health Board; 'Community Mental Health Teams', NP20 4PF
- [ ] Betsi Cadwaladr University Local Health Board; 'Mental Health Team/Psychiatry', LL18 5UJ
- [ ] Cardiff University; 'Child & Adolescent Psychiatry', CF14 7XB
- [ ] Cwm Taf Health Board; Adult Mental Health Service, CF72 8XR
- [ ] Cwm Taf Health Board; 'Royal Glamorgan Hospital Outpatient', Community Mental Health Service, CF72 8XR
- [ ] GIG Cymru NHS WALES; 'Ty Illtyd Mental Health Resource Centre', Community Mental Health Service, LD3 8AH
- [ ] MIND; National Charity, E15 4BQ
- [ ] NHS England; National Service 'Improving Access to Psychological Therapies (IAPT)', Mental Health Triage, LS2 7UE
- [ ] NHS Wales Gwynedd; 'South Gwynedd & Arfon Community Mental Health Team', LL40 1DU
- [ ] Powys Teaching Health Board; 'Bro Hafren Mental Health Resource Centre', Community Mental Health Service, SY16 2NG
- [ ] Powys Teaching Health Board; 'The Hazels Mental Health Resource Centre', Community Mental Health Service, LD1 5HF
- [ ] South London & Maudsley NHS FT; National Adult ADHD Service, SE5 8AZ

2018 CATCh-uS: Children and adolescents with Attention Deficit Hyperactivity Disorder in transition between children’s services and adult services

West Midlands: Mapping the availability of services for people aged 18 and above with ADHD

These are the services you selected. Have you, or do you know someone aged 18+ who has received treatment or support at this service for their ADHD?
If yes, please tick.

- ☐ 2gether NHS FT; 'CAMHS', HR1 2HU
- ☐ ADHD Foundation; National Charity, L2 2AH
- ☐ ADHD Smart; Private Provider, NN4 8AF
- ☐ Birmingham & Solihull MH NHS FT; '0-25 Mental Health Service', B4 6DF
- ☐ Birmingham & Solihull MH NHS FT; 'Neuropsychiatry', B15 2FG
- ☐ Birmingham Community Healthcare NHS FT; 'Learning Disability', B13 8JL
- ☐ Coventry & Warwickshire Partnership NHS Trust; 'ADHD Transitions', CV2 2TE
- ☐ Coventry & Warwickshire Partnership NHS Trust; 'Community Mental Health Teams', B37 7HJ
- ☐ Dudley & Walsall MH Partnership NHS Trust; 'Neurodevelopmental', Dudley, DY1 2LZ
- ☐ Dudley & Walsall MH Partnership NHS Trust; 'Neurodevelopmental', Walsall, WS2 9XH
- ☐ F.A.C.T; 'Fact Northants', Support Group, NN3 2JF
- ☐ MIND; National Charity, E15 4BQ
- ☐ NHS England; National Service 'Improving Access to Psychological Therapies (IAPT)', Mental Health Triage, LS2 7UE
- ☐ North Staffordshire Combined Healthcare NHS Trust; 'Learning Disability', ST4 6TH
- ☐ Northamptonshire Healthcare NHS FT; 'ADHD & Aspergers Service', NN15 7PW
- ☐ South London & Maudsley NHS FT; National Adult ADHD Service, SE5 8AZ
- ☐ South Staffordshire & Shropshire Healthcare NHS FT; Adult Mental Health Service, ST16 3SR
- ☐ Sutton Medical Consulting Centre; Private Provider, B75 6DX

2018 CATCh-uS: Children and adolescents with Attention Deficit Hyperactivity Disorder in transition between children’s services and adult services

Mapping the availability of services for people aged 18 and above with ADHD

Are there any services* missing from the previous list that you know treat or

support adults with ADHD? If YES, please complete as many details as possible below.
***A service could be NHS, private, a transition team, a charity or support group.**

Service 1

Name	
Postcode	
Town/City	
Website Link	
Other Identifying Information	

Have you, or do you know someone aged 18+ who has received treatment or support at this service for their ADHD?

- ◯ Yes
- ◯ No

Service 2

Name	
Postcode	
Town/City	
Website Link	
Other Identifying Information	

Have you, or do you know someone aged 18+ who has received treatment or support at this service for their ADHD?

- ◯ Yes
- ◯ No

Service 3

Name	
Postcode	
Town/City	
Website Link	
Other Identifying Information	

Have you, or do you know someone aged 18+ who has received treatment or support at this service for their ADHD?

- ◯ Yes
- ◯ No

CATCh-uS

2018 CATCh-uS: Children and adolescents with Attention Deficit Hyperactivity Disorder in transition between children's services and adult services

Mapping the availability of services for people aged 18 and above with ADHD

UK regions...

We want to know who is responding to our survey, so please let us know where you live*. All information will be treated confidentially, with data stored securely and only used for the purpose of mapping areas responses come from.

* What is your postcode?
(For example EX1 2LU)

* Which region of the UK are you based in?

*If you are a clinician or working with people with ADHD, please choose the postcode and region of your workplace.

Thank you!

If you would like to add anything to your answers, please comment below:

Do you want to hear about findings from this study? If so, please enter your email address below and we will send you a link to the final report (expected early 2019). We will only use your email address to share our findings with you.

Full details, including the list of ADHD services can be found on the mapping page of our website http://medicine.exeter.ac.uk/catchus/. Twitter: #catchusADHD.
Thank you for completing this survey.
