# Supplementary material for: Seven steps to mapping health service provision: lessons learned from mapping services for adults with Attention-Deficit/Hyperactivity Disorder (ADHD) in the UK
Source: BMC Health Serv Res. 2019 Jul 9;19:468. doi: 10.1186/s12913-019-4287-7 (PMC6617903; doi:10.1186/s12913-019-4287-7)
Supplement: Supplementary file 3 — 2016 Pilot Survey. (PDF 590 kb) [file 12913_2019_4287_MOESM3_ESM.pdf]

CATCh-uS: Children and adolescents with Attention Deficit Hyperactivity Disorder in transition between children's services and adult services

Mapping the availability of services for young people with ADHD

The CATCh-uS study is funded by the National Institute for Health Research and has ethical approval. Details can be found on our website. The aim of this survey is to map available services for young people with Attention Deficit Hyperactivity Disorder (ADHD) who are aged 18 and over. We only want to know about services in your area and will not share any of your personal information. The survey is anonymous and confidential. Answers will help to create a list of existing services. This is to support the overall study aims of informing and improving service provision for young adults with ADHD.

Please answer the following questions to help us map available ADHD services in the UK. The survey should take no longer than 5 minutes to complete.

1. Are you a...?

- ◯ Young person (from 14 up to 17 years old)
- ◯ Young adult (18 or older)
- ◯ A parent/carer of a young person
- ◯ A clinician working with young people and/or adults with ADHD
- ◯ Other (please specify)

CATCh-uS

CATCh-uS: Children and adolescents with Attention Deficit Hyperactivity Disorder in transition between children's services and adult services

Mapping the availability of services for young people with ADHD

2. Where do you live in the UK?

3. Which County or London Borough do you live in?

CATCh-uS: Children and adolescents with Attention Deficit Hyperactivity Disorder in transition between children's services and adult services

Mapping the availability of services for young people with ADHD

4. Do you or does a person you care for have a clinical diagnosis of ADHD?

- Yes
- No
- Awaiting a diagnosis / Undiagnosed
- Don't know
- Comments:

5. Do you know about any NHS provided or other mental health services* for people with ADHD aged 18 +?

*in your area or available to you

- Yes
- No
- Don't know
- Other (give details)

6. Which of the following sectors do you work in? (Tick all that apply)

- ◯ Private Sector
- ◯ Private Provider for NHS
- ◯ Community Interest Company (or equivalent) for NHS
- ◯ NHS
- ◯ Other (please specify)

[ ]

7. Please provide details of your job:

Job Title [ ]

Details [ ]

8. Which region of the UK do you work in?

[▾]

[ ]

9. Which County or London Borough do you work in?

[▾]

[ ]

10. For this study we would like to interview a small number of clinicians from different areas. **If you are a GP** or **clinician and you see/treat patients 18 years old or older with ADHD**, would you be willing to be contacted about taking part in a short telephone *interview? If so please provide contact details below:

*If selected, this would be about your experiences in general of transition and/or referral of young adults with ADHD into adult services.

Name	
Job Role	
Contact Telephone Number	
Email Address	

11. Where you work are there NHS provided mental health services for people aged 18 years and above with ADHD?

- ◯ Yes
- ◯ No
- ◯ Don't know

CATCh-uS

CATCh-uS: Children and adolescents with Attention Deficit Hyperactivity Disorder in transition between children's services and adult services

Mapping the availability of services for young people with ADHD

12. Please provide as many details as you can about the service* or services (either the name, the postcode or the town).

*A service could be your specialist doctor, a hospital, a clinic or a support group

Service 1	
Service 2	
Service 3	

13. Details of the service*

*This question is optional. If you don't know how to answer it then move on to the next question

	Is this service provided by	Is this service a part of
Service 1		
Service 2		
Service 3		

If you have other details please add them here:

14. Do these services offer (answer all that apply):*

*This question is optional. If you don't know how to answer it then move on to the next question

	Assessment	Diagnosis
Service 1		
Service 2		
Service 3		

Other (please specify)

15. Do these services offer (answer all that apply):*

*This question is optional. If you don't know how to answer it then move on to the next question

	Treatment (Medication)	Treatment (Other Intervention)
Service 1		
Service 2		
Service 3		

Other (please specify)

Thank you!

16. If you would like to add anything to your answers, please comment below:

Thank you for your help. A report on this study is expected in 2019 and full details will be available on the website. If you have any queries about the CATCh-uS study, please visit our website: http://medicine.exeter.ac.uk/catchus/

CATCh-uS: Children and adolescents with Attention Deficit Hyperactivity Disorder in transition between children's services and adult services

Mapping the availability of services for young people with ADHD

IF YOU ARE A PSYCHIATRIST PLEASE CONTINUE. IF YOU ARE NOT PLEASE FOLLOW THIS OTHER SURVEY LINK: https://www.surveymonkey.co.uk/r/CATCh-uS_SU.

The CATCh-uS study is funded by the National Institute for Health Research and has ethical approval. Details can be found on our website. The aim of this survey is to map available services for young people with Attention Deficit Hyperactivity Disorder (ADHD) who are aged 18 and over. The focus is on service provision and no personal or identifiable data will be shared. Data gathered will be used to create a map of current services and will feed into the overall CATCh-uS study aims of informing and improving service provision for this vulnerable group.

Please answer the following questions to help us map available ADHD services in the UK. The survey should take no longer than 5 minutes to complete.

1. Are you

- ◯ Child and Adolescent Psychiatrist
- ◯ General Adult Psychiatrist
- ◯ Trainee Registrar in Psychiatry
- ◯ Other (please specify)

2. Please indicate your grade

- ◯ Trainee
- ◯ Staff Grade / Associate Specialist / Trust Doctor
- ◯ Consultant
- ◯ Other (please specify)

3. In which of the following sectors do you work? (Tick all that apply)

- [ ] Private Sector
- [ ] Private Provider for NHS
- [ ] Community Interest Company (or equivalent) for NHS
- [ ] NHS
- [ ] Other (please specify)

4. Which region of the UK do you work in?

5. Which County or London Borough do you work in?

6. Where you work, are there NHS provided mental health services for people aged 18 years and above with ADHD?

- ( ) Yes
- ( ) No
- ( ) Don't know

CATCh-uS

CATCh-uS: Children and adolescents with Attention Deficit Hyperactivity Disorder in transition between children's services and adult services

Mapping the availability of services for young people with ADHD

7. Please provide as many details of the service or services as possible (postcode, town, website):

Service 1

Service 2

Service 3

8. Details of the service

	Is the service provided by staff	Is this service a part of
Service 1		
Service 2		
Service 3		

9. Do these services offer (answer all that apply)

	Assessment	Diagnosis	Treatment (Medication)	Treatment (Other Intervention)
Service 1				
Service 2				
Service 3				

CATCh-uS: Children and adolescents with Attention Deficit Hyperactivity Disorder in transition between children's services and adult services

Thank you!

10. For this study we would like to interview a small number of clinicians from different areas. If you see/treat patients 18 years old or older with ADHD and would be willing to take part in a short telephone *interview, please could you provide your contact details below:

*If selected, this would be about your experiences in general of transition and/or referral of young adults with ADHD into adult services.

Name

Contact Telephone Number

Email Address

11. If you would like to elaborate on any of your answers, please provide comments below:

Thank you for your help. A report on this study is expected in 2019 and full details will be available on the website. If you have any queries about the CATCh-uS study, please visit our website: http://medicine.exeter.ac.uk/catchus/.

Paediatrics - CATCh-uS: Children and adolescents with Attention Deficit Hyperactivity Disorder in transition between children's services and adult services

Mapping the availability of services for young people with ADHD

IF YOU ARE A PAEDIATRICIAN PLEASE CONTINUE. IF YOU ARE NOT PLEASE FOLLOW THIS OTHER SURVEY LINK: https://www.surveymonkey.co.uk/r/CATCh-uS_SU.

The CATCh-uS study is funded by the National Institute for Health Research and has ethical approval. **Details can be found on our website. The aim of this survey is to map available services for young people with Attention Deficit Hyperactivity Disorder (ADHD) who are aged 18 and over. The focus is on service provision and no personal or identifiable data will be shared. Data gathered will be used to create a map of current services and will feed into the overall CATCh-uS study aims of informing and improving service provision for this vulnerable group.**

Please answer the following questions to help us map available ADHD services in the UK. The survey should take no longer than 5 minutes to complete.

1. Are you

- ◯ Community Child Health Paediatrician
- ◯ General Paediatrician
- ◯ Registrar / Speciality Trainee in Paediatrics
- ◯ Paediatric Neurologist
- ◯ Paediatrician in Neurodisability
- ◯ Different Paediatric Specialist or Other (please specify)

2. Please indicate your grade

- ◯ Consultant
- ◯ Staff Grade / Associate Specialist / Trust Doctor
- ◯ Trainee
- ◯ Other (please specify)

3. In which of the following sectors do you work? (Tick all that apply)

- [ ] Private Sector
- [ ] Private Provider for NHS
- [ ] Community Interest Company (or equivalent) for NHS
- [ ] NHS
- [ ] Academia
- [ ] Other (please specify)

4. Which region of the UK do you work in?

5. Which County or London Borough do you work in?

6. Do you see children/adolescents with ADHD?

- ( ) Yes
- ( ) No
- ( ) Other (please specify)

CATCh-uS

Paediatrics - CATCh-uS: Children and adolescents with Attention Deficit Hyperactivity Disorder in transition between children's services and adult services

Mapping the availability of services for young people with ADHD

7. Where do you refer children with ADHD (with or without co-morbidities) who reach the age boundary for paediatric services on to? (tick all that apply)

- [ ] Adult Mental Health Services
- [ ] Specialist ADHD clinic or service
- [ ] Learning Disability Service
- [ ] Youth or Young People Mental Health Service
- [ ] GP
- [ ] Don't know
- [ ] No service

Other (please specify)

8. Do you have any additional comments on the question above?

Paediatrics - CATCh-uS: Children and adolescents with Attention Deficit Hyperactivity Disorder in transition between children’s services and adult services

Mapping the availability of services for young people with ADHD

9. Are there NHS provided mental health services for people aged 18 years and above with ADHD in your area?

- ( ) Yes
- ( ) No
- ( ) Don't know

Paediatrics - CATCh-uS: Children and adolescents with Attention Deficit Hyperactivity Disorder in transition between children’s services and adult services

Mapping the availability of services for young people with ADHD

10. Please provide as many details of the service or services as possible (postcode, town, website):

Service 1	
Service 2	
Service 3	

11. Details of the service

	Is the service provided by staff	Is this service a part of
Service 1		
Service 2		
Service 3		

12. Do these services offer (answer all that apply)

	Assessment	Diagnosis	Treatment (Medication)	Treatment (Other Intervention)
Service 1				
Service 2				
Service 3				

CATCh-uS

Paediatrics - CATCh-uS: Children and adolescents with Attention Deficit Hyperactivity Disorder in transition between children's services and adult services

Thank you!

13. If you would like to elaborate on any of your answers, please provide comments below:

Thank you for your help. A report on this study is expected in 2019 and full details will be available on the website. If you have any queries about the CATCh-uS study, please visit our website: http://medicine.exeter.ac.uk/catchus/

CATCh-uS: Children and adolescents with Attention Deficit Hyperactivity Disorder in transition between children's services and adult services

Mapping the availability of services for young people with ADHD

IF YOU ARE A GP PLEASE CONTINUE. IF YOU ARE NOT PLEASE FOLLOW THIS OTHER SURVEY LINK: https://www.surveymonkey.co.uk/r/CATCh-uS_SU.

The CATCh-uS study is funded by the National Institute for Health Research and has ethical approval. Details can be found on our website. The aim of this survey is to map available services for young people with Attention Deficit Hyperactivity Disorder (ADHD) who are aged 18 and over. The focus is on service provision and no personal or identifiable data will be shared. Data gathered will be used to create a map of current services and will feed into the overall CATCh-uS study aims of informing and improving service provision for this vulnerable group.

Please answer the following questions to help us map available ADHD services in the UK. The survey should take no longer than 5 minutes to complete.

1. Are you

- ◯ General Practioner
- ◯ General Practitioner Registrar
- ◯ Other (please specify)

[ ]

2. Do you have a special interest or extended role in mental health?

- ◯ Yes
- ◯ No

Comments

[ ]

3. Do you have a role in commissioning NHS services?

- ◯ Yes
- ◯ No

CATCh-uS: Children and adolescents with Attention Deficit Hyperactivity Disorder in transition between children's services and adult services

4. If yes, please provide

Name of Clinical Commissioning Group

Your job role/title within the CCG

5. In which of the following sectors do you work? (tick all that apply)

- [ ] Private Sector
- [ ] Private Provider for NHS
- [ ] Community Interest Company (or equivalent) for NHS
- [ ] NHS
- [ ] Academia
- [ ] Other (please specify)

6. Which region of the UK do you work in?

7. Which County or London Borough do you work in?

8. Are you actively involved in the management of ADHD in any of your patients (child and/or adult) (for example by prescribing)?

- ( ) Yes
- ( ) No

Comments

CATCh-uS: Children and adolescents with Attention Deficit Hyperactivity Disorder in transition between children's services and adult services

Mapping the availability of services for young people with ADHD

9. How do you manage the ADHD symptoms of children and adolescents with ADHD? (tick all that apply)

- ☐ In collaboration with Paediatric Services
- ☐ In collaboration with Child and Adolescent Mental Health Services
- ☐ In collaboration with Learning Disability Services
- ☐ In collaboration with Specialist ADHD Services
- ☐ I manage their ADHD symptoms myself, without specialist support
- ☐ I don't manage their ADHD symptoms as there is not a specialist service to collaborate with
- ☐ Other (please specify)

10. How do you manage the ADHD symptoms of young adults with ADHD who are aged 18 and above? (tick all that apply)

- ☐ In collaboration with Adult Mental Health Services
- ☐ In collaboration with a Specialist ADHD clinic or service
- ☐ In collaboration with a Learning Disability Service
- ☐ In collaboration with a Youth or Young People Mental Health Service
- ☐ I manage their ADHD symptoms myself, without specialist support
- ☐ I don't manage their ADHD symptoms as there is not a specialist service to collaborate with
- ☐ Don't know

Other (please specify)

11. Do you have any additional comments on the question above?

CATCh-uS

CATCh-uS: Children and adolescents with Attention Deficit Hyperactivity Disorder in transition between children's services and adult services

Mapping the availability of services for young people with ADHD

12. Are there NHS provided mental health services for people aged 18 years and above with ADHD in your area?

- ◯ Yes
- ◯ No
- ◯ Don't know

CATCh-uS

CATCh-uS: Children and adolescents with Attention Deficit Hyperactivity Disorder in transition between children's services and adult services

Mapping the availability of services for young people with ADHD

13. Please provide as many details of the service or services as possible (postcode, town, website):

Service 1	
Service 2	
Service 3	

14. Details of the service

	Is the service provided by staff	Is this service a part of
Service 1		
Service 2		
Service 3		

15. Do these services offer (answer all that apply)

	Assessment	Diagnosis	Treatment (Medication)	Treatment (Other Intervention)
Service 1				
Service 2				
Service 3				

CATCh-uS

CATCh-uS: Children and adolescents with Attention Deficit Hyperactivity Disorder in transition between children's services and adult services

Mapping the availability of services for young people with ADHD

16. For this study we would like to interview a small number of clinicians from different areas. If selected, would you be willing to be contacted about taking part in a short telephone interview?

*This would be about your experiences in general of transition and/or referral of young adults with ADHD into adult services.

- ◯ Yes
- ◯ No

CATCh-uS: Children and adolescents with Attention Deficit Hyperactivity Disorder in transition between children's services and adult services

Mapping the availability of services for young people with ADHD

17. Please provide your contact details

Name

Contact Telephone Number

18. Please provide your email address:

Email Address

CATCh-uS: Children and adolescents with Attention Deficit Hyperactivity Disorder in transition between children's services and adult services

Thank you!

19. If you would like to elaborate on any of your answers, please provide comments below:

Thank you for your help. A report on this study is expected in 2019 and full details will be available on the website. If you have any queries about the CATCh-uS study, please visit our website: http://medicine.exeter.ac.uk/catchus/ .
