# Supplementary material for: Seven steps to mapping health service provision: lessons learned from mapping services for adults with Attention-Deficit/Hyperactivity Disorder (ADHD) in the UK
Source: BMC Health Serv Res. 2019 Jul 9;19:468. doi: 10.1186/s12913-019-4287-7 (PMC6617903; doi:10.1186/s12913-019-4287-7)
Supplement: Supplementary file 4 — Key Research Partners. (DOCX 12 kb) [file 12913_2019_4287_MOESM4_ESM.docx]

**Key Research Partners**

| Name | Abbreviation | Website |
| --- | --- | --- |
| AADD-UK The site for and by adults with ADHD | AADD-UK | https://aadduk.org/ |
| ADHD Foundation |  | https://www.adhdfoundation.org.uk/ |
| Association for Child and Adolescent Mental Health | ACAMH | https://www.acamh.org/ |
| British Association for Community Child Health | BACCH | http://www.bacch.org.uk/index.php |
| PenCRU: CATCh-uS Parent Advisory Group |  | <http://medicine.exeter.ac.uk/catchus/> & <http://www.pencru.org/getinvolved/ourfamilyfaculty/> |
| Clinical Research Network, England | CRN | https://www.nihr.ac.uk/about-us/how-we-are-managed/managing-centres/crn/ |
| Clinical Research Network South West Peninsula | CRN SW | https://www.nihr.ac.uk/nihr-in-your-area/south-west-peninsula/ |
| Royal College of General Practitioners | RCGPs | http://www.rcgp.org.uk/ |
| Royal College of Psychiatrists | RCPsych | https://www.rcpsych.ac.uk/ |
| UK Adult ADHD Network | UKAAN | https://www.ukaan.org/ |
